# ILVES: Accurate and Efficient Bond Length and Angle Constraints in Molecular Dynamics

**DOI:** 10.1021/acs.jctc.5c01376

**Published:** 2025-09-04

**Authors:** Lorién López-Villellas, Carl Christian Kjelgaard Mikkelsen, Juan José Galano-Frutos, Santiago Marco-Sola, Jesús Alastruey-Benedé, Pablo Ibáñez, Pablo Echenique, Miquel Moretó, Maria Cristina De Rosa, Pablo García-Risueño

**Affiliations:** † Departamento de Informática e Ingeniería de Sistemas/Aragón Institute for Engineering Research (I3A), 16765 Universidad de Zaragoza, Zaragoza 50018, Spain; ‡ Department of Computing Science, Umeå University, Umeå SE-90187, Sweden; § Instituto de Biocomputación y Física de Sistemas Complejos, Zaragoza 50018, Spain; ∥ Barcelona Supercomputing Center, Barcelona 08034, Spain; ⊥ Departament d’Arquitectura de Computadors, Universitat Politècnica de Catalunya, Barcelona 08034, Spain; # Instituto de Química Física Blas Cabrera (CSIC), Madrid 28006, Spain; ∇ Istituto di Scienze e Tecnologie Chimiche “Giulio Natta” (SCITEC) − National Research Council (CNR), Milan 20133, Italy

## Abstract

All-atom, force field-based molecular dynamics simulations are essential tools in computational chemistry, enabling the prediction and analysis of biomolecular systems with atomic-level resolution. However, as system sizes and simulation time scales increase, so does the associated computational cost. To extend simulated time using the same resources, a common strategy is to constrain the fastest degrees of freedom, such as bond lengths, allowing for larger integration time steps without compromising accuracy. The de facto state-of-the-art algorithms for this purpose—SHAKE, LINCS, and P-LINCS—are integrated into most molecular dynamics packages and widely adopted across the field. Despite their impact, these methods exhibit limitations: all converge slowly when high numerical accuracy is required, and the LINCS and P-LINCS algorithms cannot handle general angular constraints, limiting further increases in time step. In this article, we introduce ILVES, a family of parallel algorithms that converge so rapidly that it is now practical to solve bond length and associated angular constraint equations as accurately as the hardware will allow. We have integrated ILVES into Gromacs, and our analysis demonstrates that it is superior to the state-of-the-art when constraining bond lengths. Due to its better convergence properties, we also show that if the time step is increased up to 3.5 fs by enforcing angular constraints, ILVES enables a 1.65× increase in simulated time using the same computational resources and wall-clock time, an outcome unattainable with current methods. This advance can significantly reduce the computational cost of most all-atom molecular dynamics simulations while improving their accuracy and extending access to larger systems and longer time scales.

## Graphical abstract

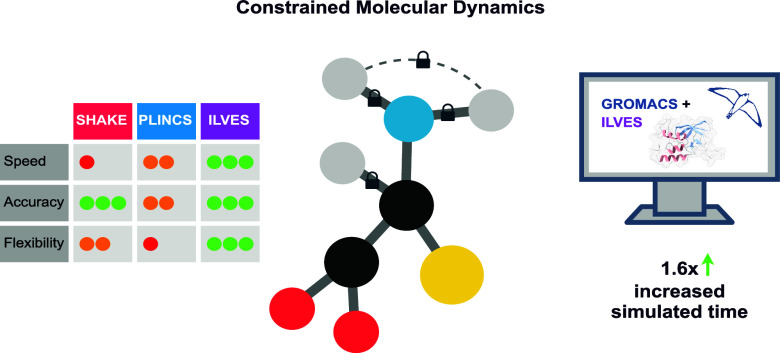

## Introduction

Molecular dynamics simulations (MD)^1,2^ have greatly impacted a wide range of fields in science and technology.^3,4^ They are of special importance in chemistry and medicine, with applications including the design of drugs and catalysts,^5–9^ e.g., helping to understand interaction- or mutation-driven biological processes.^10–12^ One of the biggest advantages of MD-based approaches is that they provide information on the simulated systems at the atomic level (positions, velocities, forces), which enables the study of phenomena whose analysis in a laboratory is often not feasible or affordable.^13,14^ Thanks to recent advances, particularly in artificial intelligence, the impact of MD is set to grow. For example, the prediction of protein structures through AlphaFold^15^ is already boosting massive-scale analysis of the behavior of proteins and their interactions for a variety of relevant areas.^16–22^

It is commonly accepted that, to be reliable, the discrete integration of the equations of motion in molecular dynamics must include at least five steps per vibration period of every degree of freedom (e.g., bond lengths, bond angles, or dihedral angles). This sets an upper limit for the value of the *time step*, i.e., for the separation between consecutive simulated times. Since the calculation at every time point requires a certain number of arithmetic operations, the size of the time step limits the total real-time that can be simulated using a given amount of computational resources. Due to this, in MD simulations it is customary to constrain some of the fastest internal degrees of freedom to fixed values. If the physical model and the resulting dynamics and thermodynamics are not distorted by doing so, the removal of the shortest vibrational periods allows an increase in the time step, thus reaching longer total times with the same computational effort.

Taking into account the well-known hierarchy that organizes vibrational periods in proteins and other biological molecules,^23^ the imposition of constraints begins with the fastest degrees of freedom and proceeds gradually to slower ones, thus allowing to increase the time step at each stage. It is very common in production MD simulations to constrain all bond lengths or those involving a hydrogen atom to increase the time step up to 2 fs. However, the situation around constraining bond angles is more heterogeneous in the literature. Although it is commonly mentioned that imposing constraints on the bond angles of hydrogen atoms is the reasonable next stage for further increasing the time step, it is difficult to find actual production simulations that do so. Instead, a variety of techniques are used, such as dummy hydrogens,^24^ redistribution of mass to make hydrogen atoms heavier and their vibrations slower,^24^ the artificial enlargement of the angular vibrational constants in the force field^23^ or the use of united atoms, i.e., the assimilation of the mass and the charge of hydrogen atoms into the heavy atoms to which they are bonded, thus effectively removing them from the model.^25^ All these techniques are useful for increasing the time step, but they introduce alterations to the model that are not justifiable a priori from physical or chemical considerations. The actual constraining of hydrogen bond angles—which is physically justifiable if we accept that this vibrational degree of freedom can be modeled as a quantum harmonic oscillator at its ground state—is performed only in a selected set of works^26–30^ and not without difficulties. This has been done, for example, using internal coordinates to integrate the equations of motion instead of Cartesian coordinates, which introduces an important computational overhead.^27^ Hydrogen bond angles have been constrained in production simulations using (P-)­LINCS, but the bond lengths of heavy atoms had to remain unconstrained due to (P-)­LINCS’ convergence problems.^31^ Finally, successful simulations with the GROMOS package have been reported using a modified version of the SHAKE algorithm to handle angles.^29^ However, no in-depth assessment of the computational cost is provided, and convergence difficulties appear in the study when a small set of new constraints is added.^29^

In this work, we present ILVES-M and ILVES-F, two parallel algorithms that solve the same system of differential-algebraic equations as SHAKE, but the constraint equations are solved using either Newton’s method or a quasi-Newton method rather than the nonlinear Gauss-Seidel method used by SHAKE. Our algorithms and software outperform the state-of-the-art algorithms, SHAKE^32^ and (P-)­LINCS.^33,34^ A review of all relevant algorithms, SHAKE, (P)-LINCS, ILVES-M, and ILVES-F, can be found in Section 2 of the Supporting Information. In particular, we show that, in most tests involving bond length constraints, the ILVES algorithms converge so rapidly that solving the constraint equations with high accuracy is not only possible but eminently practical. Most importantly, by leveraging the existing Gromacs framework, we show that ILVES-M and ILVES-F can also constrain bond angles in parallel with low computational overhead, in contrast to SHAKE and (P-)­LINCS. Our analysis shows that by constraining the bond angles of hydrogen atoms and increasing the time step to 3.5 fs, ILVES enables a 1.65× increase in simulated time using the same computational resources and wall-clock time as a simulation with the default 2 fs time step. These results establish, for the first time as far as we are aware, that constraining hydrogen bond angles enables a substantial increase in simulation throughput. The ILVES-M and ILVES-F code, integrated into Gromacs, is publicly available at https://github.com/LorienLV/_PAPER_ILVES.

## Limitations of the State-of-the-Art Constraint Solvers

SHAKE and (P-)­LINCS are decades-old algorithms. SHAKE^32^ is nearly 50 years old, while LINCS^33^ was presented in 1997 and P-LINCS^34^ appeared in 2008. Though their contribution to science has been tremendous, they have specific limitations that we seek to address. The constraint solver in the original SHAKE algorithm converges slowly and is not considered a good candidate for parallelization.^35^ Parallel versions of SHAKE^35,36^ have not been widely used, and the implementation of SHAKE in Gromacs is sequential. The Gromacs library for molecular simulation is so widely used that we have chosen it to serve as a baseline for our analysis. The successful application of (P-)­LINCS hinges on the convergence of a specific infinite series and this condition can be violated in the context of coupled angular constraints (see the Supporting Information and the original LINCS paper^33^), and the use of (P-)­LINCS for this purpose is actively discouraged in the GROMACS manual itself.^37^ An interesting example can be found in the paper^38^ where the issues were so severe that LINCS had to be abandoned in favor of SHAKE.

In general, SHAKE, LINCS, and P-LINCS are rarely used to solve the constraint equations as accurately as the hardware will allow, as this goal can only be achieved using significant time and computational resources.^39,40^ Superficially, this issue might appear insignificant, as there are many other sources of error in a simulation of molecular dynamics. However, there are cases where the error introduced in the constraints phase can result in severe distortions of the simulated system’s physics.

## Need to Solve the Constraint Equations Accurately

In a recent study,^40^ we demonstrated that solving constraints inaccurately introduces distortions that can make the simulation unreliable. Insufficient accuracy when solving the constraints is equivalent to applying undesired, spurious, and random external forces,^40^ which generates a non-negligible drift in the energy of the simulated system that consequently ruins the trustworthiness of simulations in the microcanonical (NVE) ensemble. This has led several studies to state that constraint equations must be solved *down to the limit of computational arithmetic/machine precision*.^41,42^ Simulations with a thermostat (NVT, NPT ensembles) also present such undesired energetic drifts, which contribute to making the conserved quantity (also called *conserved energy*) of the thermostat (e.g., Nosé-Hoover, V-rescale) become nonconserved. Due to this, there is no guarantee that the equations of the thermostat are satisfactorily solved, hence there is no guarantee that the simulation corresponds to the sought ensemble, which makes its reliability drop.^43,44^ Moreover, the drift introduced by the inaccurate solving of the constraints distorts the time (τ_*T*_) for reaching the sought temperature (*T*). This can be observed in the V-rescale thermostat,^44^ which calculates a rescaling factor for the velocities that, on average, is expected to make the temperature of the system approximately equal to the desired temperature *T* after a simulated time τ_*T*_ (being τ_*T*_ an input parameter of the simulation). However, due to the inaccurate constraint solving, an additional amount of energy is injected into, or extracted from, the system, which makes the average time for reaching *T* deviate from τ_*T*_ in an unknown manner. In addition, imposing constraints inaccurately systematically misestimates bond lengths and makes them randomly change their values in an irregular manner. Moreover, artifactual regimes arise as periods where the averages of the lengths of the bonds differ from the values set by constraints, which alternate with periods where the bond lengths remain nearly unchanged.^40^

In Gromacs, the default SHAKE tolerance (shake-tol, defined as the maximum relative error allowed when solving constraints) is 10^–4^. There exists no such demanded tolerance for P-LINCS, which has been said to cause unphysical dynamics and temperatures of thousands of Kelvin due to fast rotation of NH_3_ groups.^45^ Nevertheless, it is generally assumed that the average accuracy of P-LINCS with the default Gromacs parameters is typically similar to SHAKE’s. Such default settings lead to the non-negligible distorting effects on energy drifts and bond lengths mentioned above; in contrast, solving the constraints more accurately strongly dampens these undesired effects.^40^ Other research works have also found non-negligible distorting effects due to inaccurate constraint solving: ref 39. stressed that Gromacs’ default parameters lead to nonconverged results and make temperatures of the simulated system unreliable, which is fixed if constraints are accurately solved. Other research indicates that inaccuracy in constraints can lead to wrong densities^46^ or to collective motion artifacts, like spurious phase transitions from liquid to an icy state.^47^ In Section 4 of the Supporting Information, we shall argue further in favor of solving the constraint equations as accurately as the hardware will allow.

The array of inconveniences due to inaccurate constraint solving can be largely mitigated if the constraint forces are calculated with the largest possible accuracy (for the chosen numerical precision) instead of the default values in Gromacs of 10^–4^—for SHAKE—or undetermined—for LINCS—. Nevertheless, doing so has been precluded to date, most likely due to numerical complexity issues.

## ILVES Algorithms

ILVES is a family of algorithms for imposing constraints in the context of molecular dynamics. The ILVES algorithms compute discrete approximations of the solution to the same system of differential-algebraic equations as the SHAKE algorithm. However, whereas SHAKE relies on the nonlinear Gauss-Seidel method, which converges locally and linearly, the ILVES algorithms are based on Newton’s method combined with direct solvers, resulting in drastically faster convergence rates. In general, applying direct solvers to linear systems requires $O(n^{3})$ floating-point operations, being *n* the number of equations (which is equal to the number of constraints in our case^48^). Nonetheless, the particular structure of the linear systems that arise when applying constraints in MD is directly tied to the linear and sparse topology of molecular structures, so direct solvers can be applied in O(n) time for general molecules.^48,49^ The ILVES algorithms exploit this property to dramatically accelerate convergence relative to SHAKE.

In this paper, we present two algorithms, ILVES-M (“main”) and ILVES-F (“fast”), both of which leverage distributed-memory parallelism, shared-memory parallelism, and SIMD vectorization. ILVES-M solves the same system of differential-algebraic equations as SHAKE but employs Newton’s method and a direct solver. To exploit shared-memory parallelism, ILVES-M uses a custom thread-parallel *LU* factorization based on the Schur complement method.^50^ For distributed-memory parallelism, it extends this thread-parallel *LU* factorization with the Overlapping Partitioning Method (OPM).^51^ Consequently, when executed across multiple domains, ILVES-M behaves as a quasi-Newton method. However, its convergence remains extremely fast, typically requiring very few (usually zero) additional iterations compared to single-domain execution.

ILVES-F is a variant of ILVES-M that reduces the computational cost by using a fixed symmetric approximation of the coordinate matrix.^52^ The symmetry of this matrix allows for replacing *LU* factorization with *LDLT* factorization, which improves efficiency. Moreover, since the *LDLT* factorization needs only be computed once per time step, the total computational cost is nearly halved. Due to its symmetric approximation, ILVES-F behaves as a quasi-Newton method even in shared-memory executions. Nonetheless, its convergence is exceptionally fast,^53,54^ and it delivers better performance than ILVES-M in most scenarios.

In a previous article,^40^ we introduced ILVES-PC, a proof-of-concept implementation applying direct solvers and Newton’s method to calculate constraint forces in biological molecules, specifically peptides and proteins. For completeness, we include ILVES-PC in our performance analysis in this paper.

We present a detailed description of the mathematical foundations of ILVES, as well as implementation details of ILVES-M and ILVES-F, in Sections 2 and 3 of the Supporting Information.

## Results

We conducted an extensive set of simulations to assess the efficiency and reliability of ILVES, covering five representative systems: two solvated proteins (barnase, referred to as the BARN system, and the COVID-19 main protease, referred to as the COVID system), a solvated protein–DNA complex (the DNAP system), a system of 2000 benzene molecules (the BENZ system), and a tetrameric protein embedded in a lipid bilayer (the LIPID system). Full details of these systems, along with the procedures used for their preparation and simulation are provided in Sections 5 and 6 of the Supporting Information. Our reliability analysis—based on the calculation of observable quantities—as well as complementary performance results can also be found in the Supporting Information (Sections 7 and 8). Below, we summarize the outcome of our performance study, comparing ILVES-M, ILVES-F, and ILVES-PC with state-of-the-art constraint solvers. The time spent in the initialization of the solvers can be high in distributed memory simulations. For this reason, the execution times in our analysis include both initialization and processing for all solvers—except ILVES-PC, which was released as a proof of concept without distributed memory support and optimized initialization. Additionally, to ensure a fair comparison, we developed a modified version of P-LINCS that guarantees constraints are satisfied within a given tolerance, as SHAKE does; we refer to this variant as MP-LINCS. This was accomplished by repeating P-LINCS’ correction phase, controlled by the lincs-iter parameter in the original implementation until the desired maximum relative error in solving the constraints is met. Although this modification introduces additional synchronization points in parallel executions, potentially affecting performance, it is important to note that it is straightforward to modify ILVES to execute a fixed number of iterations without checking the tolerance, thereby eliminating the same synchronization points introduced in MP-LINCS. While Gromacs’ implementation of SHAKE itself is not parallelized, it can still be used in parallel simulations without domain decomposition. In such cases, SHAKE runs on a single thread, while the rest of the simulation proceeds in parallel. This approach was used to obtain the results reported for SHAKE in parallel simulations.

The speed of the constraint solving is closely related to the minimum accuracy demanded. We thus considered three values of the referred tolerance (maximum allowed relative error for every constraint): Tol = 10^–4^ (which is the default in Gromacs), Tol = 10^–8^ and Tol = 10^–12^. Simulations for Tol = 10^–4^ are performed using Gromacs compiled in single-precision mode (FP32), whereas simulations with stricter tolerances are conducted with Gromacs compiled in double-precision mode (FP64).

We have measured the performance of the algorithms in cases where constraints are imposed on either: (i) hydrogen bonds, (ii) all bonds, or (iii) all bonds together with certain angles of hydrogen atoms (specifically, H–X–H and X–O–H angles, where X represents a generic atomic species). The choice of constraint settings is generally determined—or at least recommended—by the force field used. CHARMM36 ^55^ and CHARMM36m^56^ support hydrogen bond constraints, while other force fields such as AMBER^57^ and OPLS/AA^58^ support constraints on hydrogen bonds and all bonds. Constraining angles remain uncommon, likely because none of the widely used constraint algorithms, SHAKE or (P-)­LINCS, can satisfactorily handle coupled angle constraints.

Although solving constraints is sometimes assumed to require a relatively low fraction of the total execution time of simulations, some authors inform that it can be as high as 50 or 60%.^41,59^ In our simulations constraining all bonds, SHAKE accounts for up to 92% of the total execution time, MP-LINCS up to 42%, and ILVES up to 16%. These percentages depend on the number of cores and are lower in H-bonds simulations, where SHAKE accounts for up to 60%, MP-LINCS up to 6%, and ILVES up to 5%. A detailed figure of the solvers’ relative execution times is provided in Section 8 of the Supporting Information.

Figure 1 presents the speedup over SHAKE for different numbers of threads and tasks when constraining all bonds and when constraining H-bonds. This metric is defined as the ratio of the execution time of SHAKE with a single thread to the execution time of the given solver using *N* threads. For the barnase (BARN), Covid-19 main protease (COVID), benzene (BENZ), and DNA–protein complex (DNAP), our simulations employed up to 56 threads and 1 task on a single Intel Xeon Platinum 8480+ chip. The larger (390 K atoms, 149 K ex water) lipid bilayer with proteins (LIPID) simulation was executed using up to 8 tasks and 56 threads per task, i.e., up to 4 nodes and 8 chips. The production stage of each of the simulations consisted of 50k steps of size 2 fs. In all-bonds simulations (Figure 1a), ILVES-M and ILVES-F achieve speedups over MP-LINCS across all simulations and tolerances, with a maximum of 158× over SHAKE and 14× over MP-LINCS. In H-bonds simulations (Figure 1b), MP-LINCS delivers better parallel performance than in all-bonds simulations, thus narrowing the performance gap between the solvers. In these simulations, MP-LINCS only surpasses ILVES-M and ILVES-F in the LIPID simulation at Tol = 10^–4^. On the other hand, ILVES-F delivers better performance than MP-LINCS in the rest of the simulations for a maximum speedup of 134× over SHAKE and speedups over MP-LINCS up to 1.8×.

**Figure 1:**
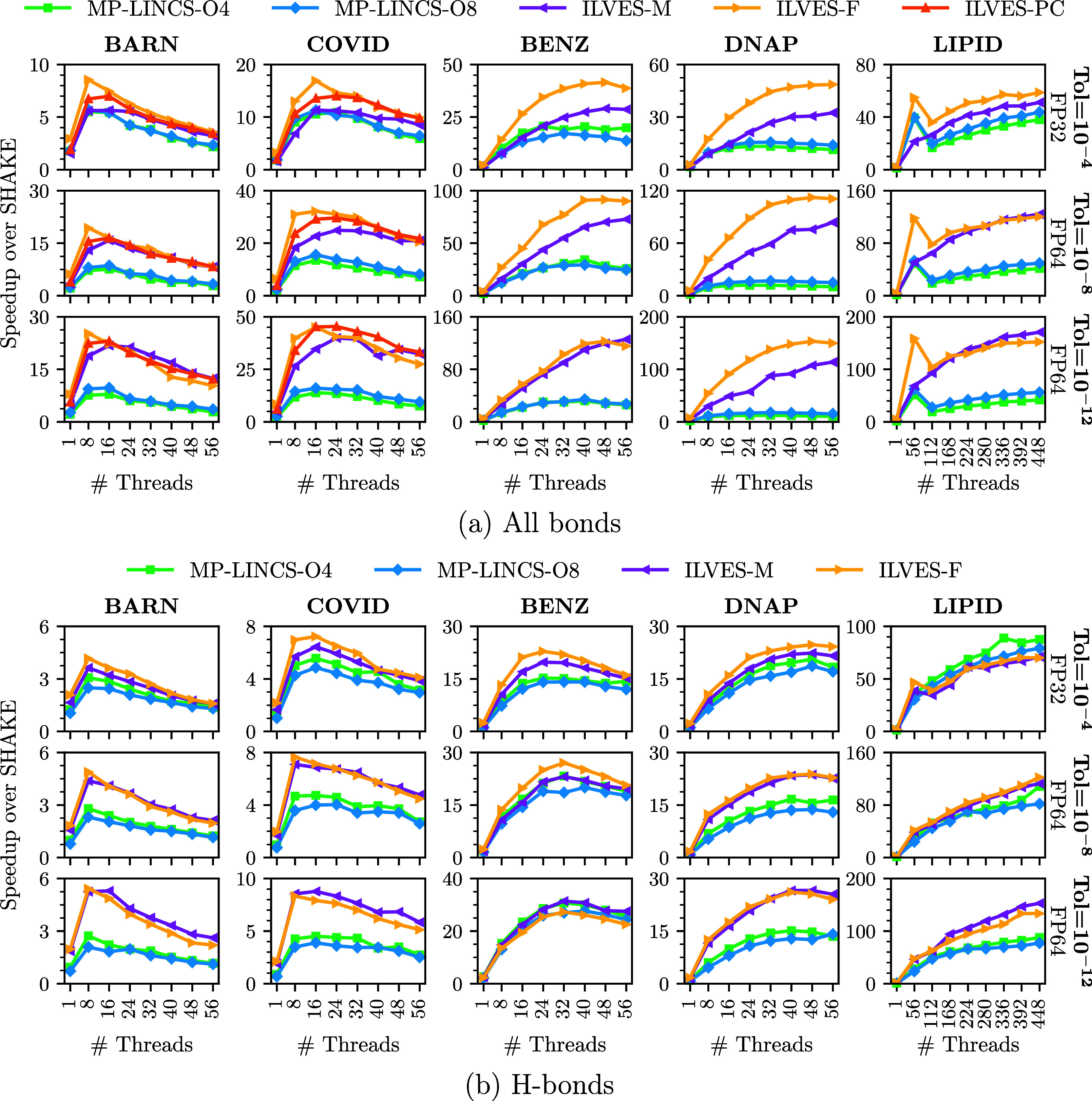
Multithread speedup over SHAKE of MP-LINCS, ILVES-M, ILVES-F, and ILVES-PC. The BARN, COVID, BENZ, and DNAP simulations are executed using a single task on a single chip of a computing node. The LIPID simulation is executed using up to 8 tasks (up to 4 nodes and 8 chips). MP-LINCS tested for lincs-order = 4 and lincs-order = 8. (a) Constraints imposed on all bonds; (b) constraints imposed on H-bonds.

Even though the tolerance defines the maximum acceptable error, the rapid convergence of the ILVES algorithm often yields errors significantly below this threshold. This results in accuracy gains, providing a compelling reason to choose ILVES over MP-LINCS in simulations where their performance is similar. In Figure 2 we display the execution time required for imposing the constraints as a function of the average relative error, which is defined as the average for *N*_*s*_ steps and *n* constraints that follows: $\left(\frac{1}{2n\cdot N_{s}}\right)\cdot \left(\sum_{k=1}^{N_{s}}\sum_{i=1}^{n}|d_{i}^{2}-(q_{a_{i}}(t_{k})-q_{b_{i}}(t_{k}){)}^{2}|/d_{i}^{2}\right)$ , where ***q***_*a*_*i*__(*t*_*k*_), ***q***_*b*_*i*__(*t*_*k*_) are the positions of both atoms joined by the *i*th constraint after applying the constraint forces corresponding to the *k*th step, and *d*_*i*_ are the bond length constants. Every point of Figure 2 corresponds to a simulation performed with different parameters (values of the constraint tolerance for SHAKE; values of the constraint tolerance or number of iterations for ILVES-M and ILVES-F; values of the number of iterations—lincs-iter—and truncation of the Neumann series—lincs-order—for P-LINCS; values of the constraint tolerance and lincs-order for MP-LINCS). The results displayed in Figure 2 correspond to the Covid main protease (4697 constraints) simulated for 50K steps in a single core. If we compare algorithms that ensure that a minimum accuracy is satisfied, like MP-LINCS-O4 and ILVES-F, we observe that, for approximately the same execution time, the latter is far more accurate than the former. For example, for constraints on all bonds, Figure 2 displays a point for MP-LINCS-O4 whose execution time is 32 s and whose average relative error is 5 · 10^–6^; it also displays a point for ILVES-F whose execution time is 27 s and whose average relative error is 9 · 10^–12^. This feature also holds for constraints on hydrogen bonds: examples of points displayed in Figure 2 are (10 s, 10^–6^) for MP-LINCS-O4, (9 s, 7 · 10^–12^) for ILVES-F and (10 s, 3 · 10^–14^) for ILVES-M. This example indicates that, for similar execution times, the ILVES algorithms are between 500,000 and 30,000,000 times more accurate than P-LINCS algorithms. Figure 2 indicates that the fast convergence of the ILVES methods makes much more accurate solutions possible requiring very low execution times, making it affordable to solve constraints near the limit of machine precision. We stress that increasing the accuracy of constraint solving is also desirable in simulations made with numerical single precision. In such cases, our tests indicate that the maximum enforceable tolerance is about Tol = 10^–6^ instead of the value Tol = 10^–12^ which corresponds to double precision.

**Figure 2:**
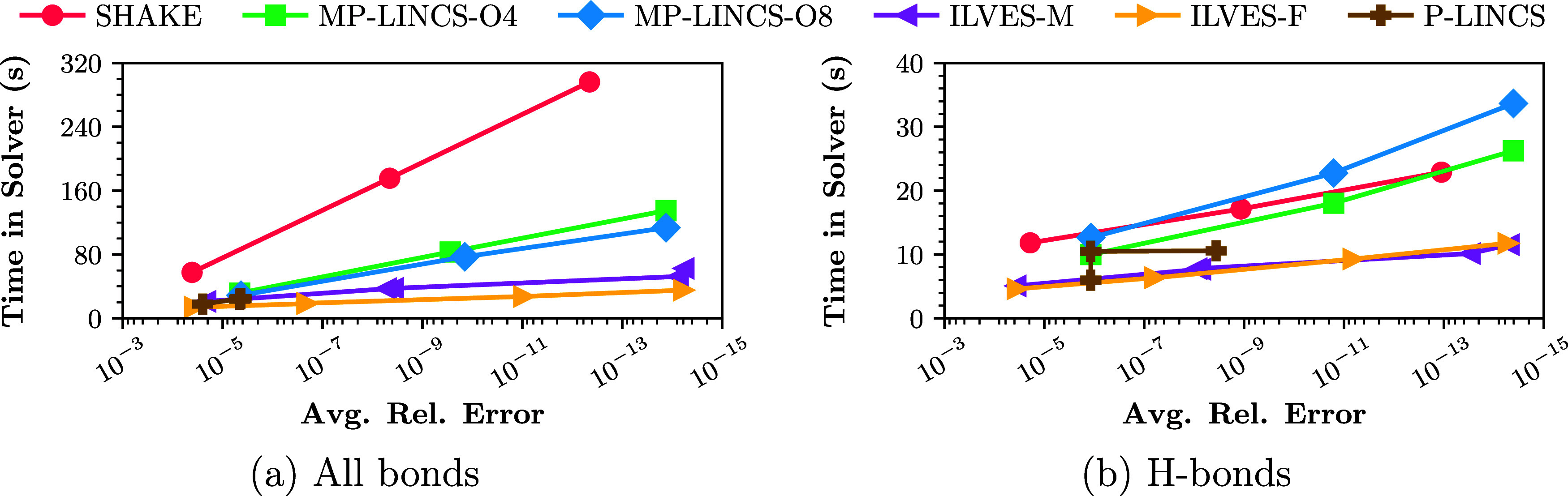
Execution time of the block for solving constraints as a function of the average relative error in satisfying them for different constraint solvers. (a) Constraints imposed on all bonds; (b) constraints imposed on H-bonds. Note that the *y*-axis is logarithmic and that the tolerance decreases from left to right.

The discussion presented in this section so far corresponds to the case of imposing constraints on just bond lengths, which limits the maximum time step to 2 fs. But, in addition to the possibility of achieving a higher degree of accuracy and computational savings that the ILVES family of algorithms provides for this very common set of constraints, its better convergence properties also allow us to cross a line unprecedented in the literature as far as we are aware. In what follows, we demonstrate that ILVES can be utilized to efficiently impose constraints on specific bond angles of hydrogen atoms, thereby enabling an increase in both the time step and the simulation throughput.

At present Gromacs offers time steps beyond 2 fs by applying several techniques such as *mass repartitioning* or *virtual sites*.^30,37^ Mass repartitioning involves assigning hydrogen atom masses greater than 1 atomic mass unit, which is compensated by withdrawing part of the mass of heavy atoms. Virtual sites consist of determining the position of hydrogen atoms as a function of the position of three nearby heavy atoms, i.e., without applying forces to the hydrogen atoms. Although these approaches have been shown to produce suitable results for some observables and thermodynamic quantities,^60^ the additional assumptions that they introduce in the physical model can significantly alter some kinetic properties such as diffusion in lipid membranes or the typical time of protein–ligand binding.^61–63^ By comparison, imposing constraints on H-angles is chemically and physically justifiable, as quantum harmonic oscillators resemble constraints more closely than classical harmonic oscillators. Literature indicates that the time step can be safely increased up to 4 fs by constraining all covalent bonds and the angles involving hydrogen atoms.^23,24,27,29^ However, the option constraints = h-angles in Gromacs only imposes constraints on a subset of all the bond angles related to hydrogen atoms, namely those defined between two hydrogen atoms connected to the same heavy atom X in a H-X-H scheme and the angle between a hydrogen atom connected to an oxygen atom and the heavy atom X connected to the oxygen in a X–O-H scheme. This freezes some of the vibrations associated with the angular degrees of freedom of hydrogen atoms, but not all of them, and this is the reason why constraints = h-angles in Gromacs allows an increase of the time step to 3.5 fs but not to 4 fs. The implementation of full bond angle constraints will be a matter of future research.

Despite its availability, the constraints = h-angles option of Gromacs has not been successfully used in the literature to increase the time step, most likely due to the limitations of the state-of-the-art constraint solvers: (P-)­LINCS is usually unable to impose constraints on coupled angles,^38^ and SHAKE converges extremely slowly.^64^ This is shown in Figure 3, in which we increase the time step of the LIPID simulation to 3.5 fs (this is the identified upper bound for stable simulations when using the constraints = h-angles setup). The referred figure reports the simulation performance in nanoseconds simulated per day and the percentage of execution time spent on the constraint solver using SHAKE, P-LINCS, ILVES-M, and ILVES-F, under two configurations: the default Gromacs settings (constraints = h-bonds, ts = 2 fs, Tol = 10^–4^) and the new settings (constraints = h-angles, ts = 3.5 fs, Tol = 10^–4^). The simulation was performed using all 56 cores of an Intel Xeon Platinum 8480+ processor and ran for 1.5 million steps. The results show that increasing the time step from 2 to 3.5 fs by introducing angle constraints causes SHAKE to dominate the simulation time, accounting for 93% of the total runtime and severely limiting overall performance. In addition, P-LINCS does not work with constraints = h-angles (marked as N/A in the figure). In contrast, ILVES enables the simulation to run significantly faster, increasing performance from 23 ns/day to 38 ns/day, which translates to a 1.65× improvement. Furthermore, ILVES accounts for a small fraction of the total runtime: 14% for ILVES-M and 7% for ILVES-F.

**Figure 3:**
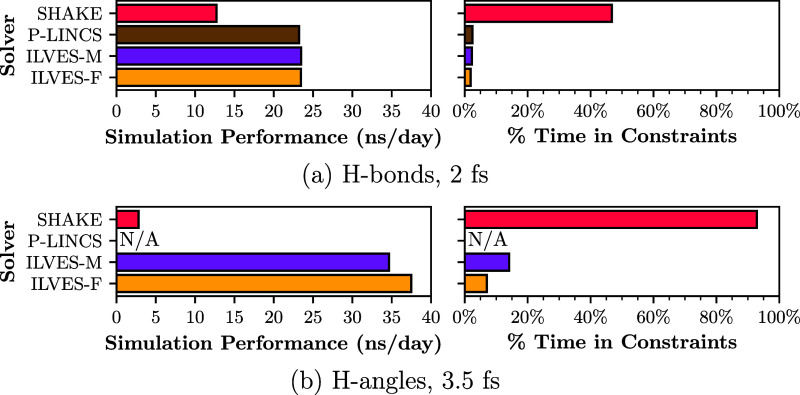
LIPID simulation performance (left) and percentage of the execution time spent on the constraint solver (right) using a 2 fs time step with constraints = h-bonds (a) and a 3.5 fs time step with constraints = h-angles (b), across four constraint solvers: SHAKE, P-LINCS, ILVES-M, and ILVES-F. The performance is reported in nanoseconds simulated per day, using all 56 cores of an Intel Xeon Platinum 8480+ chip. P-LINCS is not compatible with constraints = h-angles and is marked as N/A in the figure.

The previous results demonstrate that ILVES paves the way for a new approach to increasing the time step without relying on potentially unphysical approximations, such as mass repartitioning. Nevertheless, further research is required. Currently, increasing the time step beyond 3.5 fs is not possible within Gromacs, as it lacks support for constraining a larger set of angles involving hydrogen atoms. Additionally, existing force fields have not been parametrized for use with angle constraints. Further investigation is, therefore, necessary to establish how to correctly impose angle constraints—and potentially dihedral constraints—so that ILVES can enable time steps well above 4 fs and to evaluate how introducing such constraints would affect the physical accuracy and stability of molecular dynamics simulations.

## Conclusions and Future Work

In this work, we introduce novel parallel algorithms based on Newton’s method and direct linear solvers, designed to impose constraints on molecular systems. These algorithms demonstrate significant improvements in both accuracy and efficiency compared to the current state-of-the-art methods when constraining bond lengths. Moreover, they enable the efficient constraining of additional degrees of freedom in parallel, establishing the foundation for increasing the time step of simulations. We show that when the time step is increased from 2 to 3.5 fs by constraining some hydrogen angles (leveraging Gromacs’ existing framework for this task), P-LINCS does not work, and SHAKE dominates the total execution time of Gromacs, significantly degrading performance. In contrast, our solvers enable a 1.65× increase in simulated time using the same computational resources and wall-clock time.

In this article, we have shown that increasing the time step by applying angle constraints in combination with ILVES can yield substantial performance gains. Looking toward future research, we plan to investigate how to further and reliably extend the time step by constraining all hydrogen angles,^31^ dihedral angles,^28^ and other internal degrees of freedom. ILVES is expected to solve the equations associated with these additional constraints efficiently, thus enabling higher time steps in simulations with and without virtual sites,^30^ which we also intend to explore. Further work will also focus on integrating these constraints into molecular dynamics packages and assessing their effects on simulation accuracy and stability. The current implementation of P-LINCS found in Gromacs supports GPU execution when synchronization between threads or nodes is not required, such as when constraining hydrogen bonds. We are developing GPU-accelerated versions of ILVES-M and ILVES-F for these same use cases, which we expect will enable higher accuracy without increasing execution time, consistent with the CPU-based results presented in this work. Further planned research will consist of optimizing ILVES for water molecules,^65^ which—from preliminary tests—is expected to improve performance over the widely used SETTLE algorithm.^66^

## Supplementary Material



## Notes

Overview of constraints solvers for molecular dynamics, mathematical background, description of the ILVES implementation, extended study of the importance of accuracy when solving constraints, experimental setup, reliability study, and extended performance analysis (PDF)

## ○

Certest Biotec S.L., 50840, San Mateo de Gállego, Zaragoza, Spain
