# Supplementary material for: ILVES: Accurate and Efficient Bond Length and Angle Constraints in Molecular Dynamics
Source: J Chem Theory Comput. 2025 Sep 4;21(18):8711–9. doi: 10.1021/acs.jctc.5c01376 (PMC12461920; doi:10.1021/acs.jctc.5c01376)
Supplement: Supplementary file 1 [file ct5c01376_si_001.pdf]

# ILVES: Accurate and Efficient Bond Length and Angle Constraints in Molecular Dynamics

## Supplementary Materials

Lorién López-Villellas,[*,†] Carl Christian Kjelgaard Mikkelsen,[‡] Juan José Galano-Frutos,[¶,@] Santiago Marco-Sola,[§,‖] Jesús Alastruey-Benedé,[†] Pablo Ibáñez,[†] Pablo Echenique,[⊥] Miquel Moretó,[§,‖] Maria Cristina De Rosa,[#] and Pablo García-Risueño[*,¶]

†*Departamento de Informática e Ingeniería de Sistemas / Aragón Institute for Engineering Research (I3A), Universidad de Zaragoza, 50018, Zaragoza, Spain*

‡*Department of Computing Science, Umeå University, SE-90187, Umeå, Sweden*

¶*Instituto de Biocomputación y Física de Sistemas Complejos, 50018, Zaragoza, Spain*

§*Barcelona Supercomputing Center, 08034, Barcelona, Spain*

‖*Departament d'Arquitectura de Computadors, Universitat Politècnica de Catalunya, 08034, Barcelona, Spain*

⊥*Instituto de Química Física Blas Cabrera (CSIC), 28006, Madrid, Spain*

#*Istituto di Scienze e Tecnologie Chimiche "Giulio Natta" (SCITEC) – National Research Council (CNR), 20133, Milan, Italy*

@*Present address: Certest Biotec S.L., 50840, San Mateo de Gállego, Zaragoza, Spain*

E-mail: lorien.lopez@unizar.es; risueno@unizar.es

**Abstract**

This document presents relevant information about the ILVES algorithms, which do not appear in the main article due to space limitations. It is divided into three parts: theory, methods, and results. In the former (Theory), we present an overview of the constraint solvers for molecular dynamics, the mathematical background of constrained molecular dynamics, a complete derivation of the equations behind ILVES, as well as the concepts behind its implementation. Additionally, we provide a more in-depth discussion on why constraints should be solved as accurately as possible. In the second part (Methods), we present detailed information about the infrastructure on which our simulations were performed and the input data. Finally, in the third part (Results), we present calculations of observable quantities that prove that ILVES does not distort the dynamics more than SHAKE or LINCS, as well as extended outcomes about ILVES' performance.


# Table of Contents


**Part A: Theory**

1. Overview of Constraint Solvers for Molecular Dynamics S4
2. Mathematical Foundations of Constrained Molecular Dynamics S5
3. Extended Description of ILVES-M and ILVES-F S13
4. Further Discussion on the Need to Solve the Constraint Equations Accurately S19

**Part B: Methods**

5. Experimental Setup S27
6. Simulations S29

**Part C: Results**

7. Reliability Study S32

8. Extended Performance Results S36

# 1. Overview of Constraint Solvers for Molecular Dynamics

The most popular algorithms for imposing holonomic constraints in the context of molecular dynamics are decades old: SHAKE was presented in 1977, while LINCS was presented in 1997; its parallel version (P-LINCS) appeared in 2008. Apart from SHAKE, LINCS and P-LINCS, several algorithms to impose constraints on molecules have been proposed[1–10]. Among them, we highlight the profusely cited RATTLE[1] and SETTLE[2]. The former enforces constraints for both positions and velocities, while the latter imposes constraints on water molecules, treating them as rigid bodies. Other ones are typically slower than SHAKE[4] or have quadratic ($\mathcal{O}(n^2)$) or cubic ($\mathcal{O}(n^3)$) complexity[5,6,8,9].

Some proposed constraint algorithms[9,11,12] rely on direct (*analytical*) solvers[13] for the involved linear systems of equations: M-SHAKE[9] performs Gaussian elimination without applying sparse techniques, resulting in a cubic ($\mathcal{O}(n^3)$) numerical complexity in the number of constraints; MILC-SHAKE and MILCH-SHAKE [11,12] are only applicable to linear chains and n-alkanes, respectively. Application of sparse libraries for solving constraints, as well as parallel versions of SHAKE[14,15] have been proposed in the past[16], yet not widely applied. Further algorithms have been designed to impose constraints on bond angles[8,17,18] and dihedral angles[19,20]. Refs.[17,18] use more sophisticated expressions than distance constraints for angular constraints but solve the equations one after the other (i.e. *à la SHAKE*, solving the first equation first, then the second equation, etc.). This approach converges slowly and is challenging to parallelize. The execution time of $\theta$-SHAKE[17] has the same order of magnitude as SHAKE's, which is significant for constraints on bond angles. Moreover, its efficiency relies on the low connectivity of the bonds of the tackled molecules. Though there are exceptions[21–26], bond angles are not frequently constrained in MD simulations. This is likely due to the slowness of SHAKE in performing the corresponding calculations.

# 2. Mathematical Foundations of Constrained Molecular Dynamics

In this section, we present the mathematical fundamentals of constrained MD. We explicitly state the SHAKE, (P-)LINCS and ILVES algorithms.

## 2.1 Notation

We begin by defining our notation. Vectors are written using bold lowercase letters, e.g., $\boldsymbol{x}$, while matrices are written using bold uppercase letters, e.g., $\boldsymbol{A}$. If $\boldsymbol{A} = [a_{ij}]$ is any matrix, then the transpose matrix $\boldsymbol{A}^T$ is the matrix $\boldsymbol{B} = [b_{ij}]$ given by $b_{ij} = a_{ji}$. By default, all vectors are column vectors and when a row vector is needed a column vector is explicitly transposed. The Euclidean inner product $s = \boldsymbol{x} \cdot \boldsymbol{y}$ between two vectors $\boldsymbol{x} = (x_1, x_2, \ldots, x_n)^T \in \mathbb{R}^n$ and $\boldsymbol{y} = (y_1, y_2, \ldots, y_n)^T \in \mathbb{R}^n$ is given by $s = \boldsymbol{x}^T\boldsymbol{y} = \sum_{j=1}^n x_i y_i$. The corresponding Euclidean norm $\|\boldsymbol{x}\|_2$ is the non-negative real number given by $\|\boldsymbol{x}\|_2 = \sqrt{\boldsymbol{x}^T\boldsymbol{x}} = \sqrt{\sum_{j=1}^n x_j^2}$. If the function $\boldsymbol{f} = (f_1, f_2, \ldots, f_l)^T : \mathbb{R}^n \to \mathbb{R}^l$ is differentiable, then the Jacobian $\boldsymbol{F}(\boldsymbol{x})$ of $\boldsymbol{f}$ at the point $\boldsymbol{x} \in \mathbb{R}^n$ is the matrix $\boldsymbol{A} = [a_{ij}] \in \mathbb{R}^{l \times n}$ of first order partial derivatives, i.e.,

$$a_{ij} := \frac{\partial f_i}{\partial x_j}(\boldsymbol{x}) \, . \tag{1}$$

In a system of $s$ atoms, let $m_i > 0$ denote the mass of the $i$th atom and let $\boldsymbol{m}_i \in \mathbb{R}^3$ and the diagonal mass matrix $\boldsymbol{M} \in \mathbb{R}^{3s \times 3s}$ be given by

$$\boldsymbol{m}_i := (m_i, m_i, m_i)^T, \quad \boldsymbol{M} := \mathrm{diag}(\boldsymbol{m}_1^T, \boldsymbol{m}_2^T, \ldots, \boldsymbol{m}_n^T) \, . \tag{2}$$

In addition, let $\boldsymbol{q}_i, \boldsymbol{v}_i \in \mathbb{R}^3$ denote the position and velocity of the $i$th atom, and let $\boldsymbol{f}_i \in \mathbb{R}^3$ represent the force acting on it (due to any atom or field). The vectors $\boldsymbol{q}, \boldsymbol{v}, \boldsymbol{f} \in \mathbb{R}^{3s}$, which

gather all positions, velocities, and forces are defined as follows

$$\boldsymbol{q} := (\boldsymbol{q}_1^T, \boldsymbol{q}_2^T, \ldots, \boldsymbol{q}_s^T)^T\,; \quad \boldsymbol{v} := (\boldsymbol{v}_1^T, \boldsymbol{v}_2^T, \ldots, \boldsymbol{v}_s^T)^T\,; \quad \boldsymbol{f} := (\boldsymbol{f}_1^T, \boldsymbol{f}_2^T, \ldots, \boldsymbol{f}_s^T)^T\,. \tag{3}$$

Throughout this work, $s$ is the number of atoms (hence, the total number of Cartesian coordinates for positions is $3s$) and $n$ is the number of imposed constraints.

## 2.2 Equations of motion

The phenomena of interest for MD span a wide range of time scales. The subset that can be simulated is limited by the time step ($\Delta t$), which is defined as the gap between consecutive simulated times; for force fields-based MD $\Delta t$ this is typically in the range of femtoseconds (fs). Therefore, setting it to values that are as high as possible (without distorting the dynamics of the simulated system) is a major task. To accomplish this, a widely used method involves imposing constraints on the molecular internal degrees of freedom. Numerous algorithms to perform the corresponding calculations have been proposed, with SHAKE[27], LINCS[28], and P-LINCS[29] being the most frequent ones to be applied to molecules other than water.

Molecular dynamics is based on Newton's second law, which can be written as follows for a system consisting of $s$ atoms:

$$\dot{\boldsymbol{q}}(t) = \boldsymbol{v}(t)\,, \tag{4a}$$

$$\boldsymbol{M}\dot{\boldsymbol{v}}(t) = \boldsymbol{f}(t)\,, \tag{4b}$$

where $\boldsymbol{q}$, $\boldsymbol{v}$, $\boldsymbol{f} \in \mathbb{R}^{3s}$ are the vectors of the atomic positions, atomic velocities and total forces acting on the atoms; $\boldsymbol{M}$ is the matrix of (constant) atomic masses and the dot on top indicates time ($t$) derivative.

If we impose a set of $n$ bond constraints on the system (4) then the equations of motion

change to a system of differential-algebraic equations:

$$\dot{\boldsymbol{q}}(t) = \boldsymbol{v}(t)\,, \tag{5a}$$

$$\boldsymbol{M}\dot{\boldsymbol{v}}(t) = \boldsymbol{f}_{\text{ext}}(t) - \boldsymbol{G}(\boldsymbol{q}(t))^T\boldsymbol{\lambda}(t)\,, \tag{5b}$$

$$\boldsymbol{g}(\boldsymbol{q}(t)) = \boldsymbol{0}\,. \tag{5c}$$

Here $\boldsymbol{f}_{\text{ext}}$ is the total external force acting on the atoms (which can be calculated using a force field); $\boldsymbol{g} : \mathbb{R}^{3s} \to \mathbb{R}^n$ is the constraint function

$$\boldsymbol{q} \to (g_1(\boldsymbol{q}), g_2(\boldsymbol{q}), \ldots, g_n(\boldsymbol{q})) \tag{6}$$

and $\boldsymbol{G}(\boldsymbol{q}) \in \mathbb{R}^{n\times 3s}$ is the Jacobian of $\boldsymbol{g}$ with respect to the vector $\boldsymbol{q}$ of atomic coordinates. If the $i$th constraint is a bond length constraint between atoms $a_i$ and $b_i$, then we may choose

$$g_i(\boldsymbol{q}) = \frac{1}{2}\left(d_i^2 - \|\boldsymbol{q}_{a_i} - \boldsymbol{q}_{b_i}\|^2\right). \tag{7}$$

where $d_i$ (commonly a constant) is the desired distance between the atoms $a_i$ and $b_i$. This particular choice ensures that the constraint function is infinitely differentiable at every point $\boldsymbol{q}$.

The vast majority of computer simulations rely on discrete time steps. Among the standard integrators used to update positions (whether strictly following Newton's second law or a modification of it) are the Verlet[30] algorithms (e.g., Leapfrog and Velocity Verlet), as well as the Stochastic Dynamics (SD) and Langevin Dynamics algorithms[31].

Below (Secs. 2.3 and 2.4), we present a summary of the state-of-the-art algorithms for constrained MD (of molecules other than water), i.e., SHAKE, ILVES, and (P-)LINCS.

## 2.3 The SHAKE/ILVES algorithms

The SHAKE and ILVES algorithms use Verlet integration to solve the same system of differential-algebraic equations. They solve the constraint equations using different techniques. However, if the constraint equations are solved exactly and if all calculations are done without rounding errors, then they will return the same results.

The two algorithms use a pair of staggered uniform grids with fixed time step $h$ (being $h := \Delta t$). The central equations can be stated as follows

$$\boldsymbol{v}_{k+1/2} = \boldsymbol{v}_{k-1/2} + h\boldsymbol{M}^{-1}\big(\boldsymbol{f}_{ext}(t_k) - \boldsymbol{G}(\boldsymbol{q}_k)^T\boldsymbol{\lambda}_k\big)\,, \tag{8a}$$

$$\boldsymbol{q}_{k+1} = \boldsymbol{q}_k + h\boldsymbol{v}_{k+1/2}\,, \tag{8b}$$

$$\boldsymbol{g}(\boldsymbol{q}_{k+1}) = \boldsymbol{0}\,, \tag{8c}$$

where $k \in \mathbb{N}$ is the time index, i.e.,

$$t_k = kh, \quad t_{k+\frac{1}{2}} = \left(t + \frac{1}{2}\right) h$$

and

$$\boldsymbol{q}_k \approx \boldsymbol{q}(t_k), \quad \boldsymbol{v}_{k+\frac{1}{2}} \approx \boldsymbol{v}\left(t_{k+\frac{1}{2}}\right). \tag{9}$$

Equation (8c) is usually a non-linear equation for the unknown vector of Lagrange multipliers $\boldsymbol{\lambda}_k$, namely $\boldsymbol{g}(\boldsymbol{\phi}_k(\boldsymbol{\lambda})) = \boldsymbol{0}$ where $\boldsymbol{\phi}_k$ is a function obtained by combining (8a), (8b):

$$\boldsymbol{\phi}_k(\boldsymbol{\lambda}) := \boldsymbol{q}_k + h\left(\boldsymbol{v}_{k-\frac{1}{2}} + h\boldsymbol{M}^{-1}\big(\boldsymbol{f}_{ext}(t_k) - \boldsymbol{G}(\boldsymbol{q}_k)^T\boldsymbol{\lambda}\big)\right). \tag{10}$$

The SHAKE algorithm solves the nonlinear constraint equations using the nonlinear Gauss-Seidel iteration. This method converges slowly and locally, subject to very modest conditions; see[32,33] and the references therein. In contrast, the ILVES algorithms use either Newton's method or a quasi-Newton method to solve the constraint equations rapidly. We

now begin our exposition of the ILVES algorithms by stating Newton's method in the general case. Let $\boldsymbol{f} : \mathbb{R}^n \rightarrow \mathbb{R}^n$ be a differentiable function and consider the problem of solving the following system of equations:

$$\boldsymbol{f}(\boldsymbol{x}) = \mathbf{0}, \tag{11}$$

with respect to $\boldsymbol{x} \in \mathbb{R}^n$. If the Jacobian $\boldsymbol{F}(\boldsymbol{x})$ of $\boldsymbol{f}$ at any point $\boldsymbol{x}$ is nonsingular, then Newton's method is given by

$$\boldsymbol{F}(\boldsymbol{x}_l)\boldsymbol{z}_l = \boldsymbol{f}(\boldsymbol{x}_l)\,, \tag{12a}$$

$$\boldsymbol{x}_{l+1} = \boldsymbol{x}_l - \boldsymbol{z}_l\,. \tag{12b}$$

where the initial approximation $\boldsymbol{x}_0$ must be chosen by the user. In general, Newton's method will converge locally and quadratically to a zero $\boldsymbol{z}$ of $\boldsymbol{f}$ if the Jacobian $\boldsymbol{F}(\boldsymbol{z})$ at $\boldsymbol{z}$ is nonsingular, i.e., there exists a constant $C$ such that

$$\frac{\|\boldsymbol{z} - \boldsymbol{x}_{l+1}\|_2}{\|\boldsymbol{z} - \boldsymbol{x}_l\|_2^2} \rightarrow C, \quad l \rightarrow \infty, \quad l \in \mathbb{N}. \tag{13}$$

for all choices of $\boldsymbol{x}_0$, provided that $\|\boldsymbol{z} - \boldsymbol{x}_0\|_2$ is sufficiently small. We shall now state Newton's method for the non-linear constraint equation (8c). We first use the chain rule of differentiation to compute the Jacobian of the function $\boldsymbol{\lambda} \rightarrow \boldsymbol{g}(\boldsymbol{\phi}_k(\boldsymbol{\lambda}))$ and find that

$$\frac{\partial}{\partial \boldsymbol{\lambda}}(\boldsymbol{g} \circ \boldsymbol{\phi}_k)(\boldsymbol{\lambda}) = \boldsymbol{G}(\boldsymbol{\phi}_k(\boldsymbol{\lambda}))\frac{\partial \boldsymbol{\phi}_k}{\partial \boldsymbol{\lambda}}(\boldsymbol{\lambda}) = -h^2\boldsymbol{G}(\boldsymbol{\phi}_k(\boldsymbol{\lambda}))\boldsymbol{M}^{-1}\boldsymbol{G}(\boldsymbol{q}_k)^T \tag{14}$$

which is why we define the matrix function $\boldsymbol{A} : \mathbb{R}^{3s} \times \mathbb{R}^{3s} \rightarrow \mathbb{R}^{n \times n}$ given by

$$\boldsymbol{A}(\boldsymbol{x}, \boldsymbol{y}) := -h^2\boldsymbol{G}(\boldsymbol{x})\boldsymbol{M}^{-1}\boldsymbol{G}(\boldsymbol{y})^T\,. \tag{15}$$

In general, each matrix $\boldsymbol{A}(\boldsymbol{x}, \boldsymbol{y}) = [a_{ij}]$ is both sparse and *structurally* symmetric because $a_{ij} = a_{ji} = 0$ if the $i$th and $j$th bond do not share an atom[34]. However, we cannot expect

that $\boldsymbol{A}$ is symmetric in the sense that $\boldsymbol{A} = \boldsymbol{A}^T$, unless, of course, $\boldsymbol{x} = \boldsymbol{y}$.

Newton's method for solving equation (8c) with respect to the Lagrange multipliers $\boldsymbol{\lambda}_k$ can be written as

$$\boldsymbol{A}\big(\boldsymbol{\phi}_k(\boldsymbol{\lambda}_{k,l}), \boldsymbol{q}_k\big)\boldsymbol{z}_{k,l} = \boldsymbol{g}\big(\boldsymbol{\phi}_k(\boldsymbol{\lambda}_{k,l})\big), \tag{16a}$$

$$\boldsymbol{\lambda}_{k,l+1} = \boldsymbol{\lambda}_{k,l} - \boldsymbol{z}_{k,l}, \tag{16b}$$

It is common to initialize the search for $\boldsymbol{\lambda}_k$ using $\boldsymbol{\lambda}_{k,0} = \boldsymbol{0}$ and we have used this value to good effect. We emphasize that equation (16a) is a *linear* equation with respect to the unknown vector $\boldsymbol{z}_{k,l}$ and the solution of several such systems is usually required to advance the simulation from time $t_k$ to time $t_{k+1}$.

The ILVES algorithms solve these linear systems by approximating the local action of the inverse of $\boldsymbol{A}$ using a different approximation for every processing unit. The technical details are provided in Section 3.

## 2.4 The (P-)LINCS algorithms

The P-LINCS algorithm is a parallel version of LINCS. In the absence of any rounding errors, they produce identical output. The (P-)LINCS algorithms are also based on Verlet integration and use the same pair of staggered grids with a uniform time step $h$ as SHAKE and ILVES. The (P-)LINCS algorithms apply to bond constraints functions $\boldsymbol{g} : \mathbb{R}^{3N} \rightarrow \mathbb{R}^n$ where the individual components $g_i$ of $\boldsymbol{g}$ must have the form

$$g_i(\boldsymbol{q}) = \|\boldsymbol{q}_{a_i} - \boldsymbol{q}_{b_i}\|_2 - d_i, \quad i = 1, 2, \ldots, n. \tag{17}$$

Here $d_i$ is the length of the bond between atoms $a_i$ and $b_i$ whose coordinates are $\boldsymbol{q}_{a_i}$ and $\boldsymbol{q}_{b_i}$ respectively. The Jacobian $\boldsymbol{G}$ of this specific choice of constraints satisfy the elementary

identity

$$\forall\, \boldsymbol{q}, \boldsymbol{s} \in \mathbb{R}^{3s} \;:\; (G(\boldsymbol{q})\boldsymbol{s})_i = \left( \frac{\boldsymbol{q}_{a_i} - \boldsymbol{q}_{b_i}}{\|\boldsymbol{q}_{a_i} - \boldsymbol{q}_{b_i}\|} \right)^T (\boldsymbol{s}_{a_i} - \boldsymbol{s}_{b_i}), \quad i = 1, 2, \ldots, n, \tag{18}$$

and this result is used repeatedly to derive the algorithm[28]. We shall now summarize the LINCS algorithm. Given the current positions $\boldsymbol{q}_k$ and velocities $\boldsymbol{v}_{k-\frac{1}{2}}$ we compute the next vectors $\boldsymbol{q}_{k+1}$ and $\boldsymbol{v}_{k+\frac{1}{2}}$ in the following manner. An initial approximation $\boldsymbol{q}^*_{k+1}$ is computed using

$$\boldsymbol{q}^*_{k+1} = \boldsymbol{q}_k + h\boldsymbol{v}_{k-\frac{1}{2}} + h^2 \boldsymbol{M}^{-1} \boldsymbol{f}_k. \tag{19}$$

where $\boldsymbol{f}_k = \boldsymbol{f}_{\text{ext}}(\boldsymbol{q}_k)$ is the total external force acting on the atoms at the current positions $\boldsymbol{q}_k$. This definition corresponds to a single step of the Verlet method for the differential equation

$$\dot{\boldsymbol{q}}(t) = \boldsymbol{v}(t), \tag{20}$$

$$\boldsymbol{M}\dot{\boldsymbol{v}}(t) = \boldsymbol{f}_{\text{ext}}(t). \tag{21}$$

In general, one cannot expect that $\boldsymbol{q}^*_{k+1}$ solves the constraint equation $\boldsymbol{g}(\boldsymbol{q}) = \boldsymbol{0}$, which is why LINCS performs at least one additional step. Specifically, the algorithm computes

$$\boldsymbol{q}^0_{k+1} = \boldsymbol{q}^*_{k+1} - \boldsymbol{T}_k \left( \boldsymbol{G}_k \boldsymbol{q}^*_{k+1} - \boldsymbol{d} \right) \tag{22}$$

where the central matrix $\boldsymbol{T}_k$ and the vector $\boldsymbol{d}$ are given by

$$\boldsymbol{T}_k = \boldsymbol{M}^{-1} \boldsymbol{G}_k^T (\boldsymbol{G}_k \boldsymbol{M}^{-1} \boldsymbol{G}_k^T)^{-1}, \tag{23}$$

$$\boldsymbol{d} = (d_1, d_2, \ldots, d_n)^T, \tag{24}$$

and then (optionally) continues with the iteration

$$\boldsymbol{q}^{l+1}_{k+1} = \boldsymbol{q}^l_{k+1} - \boldsymbol{T}_k \left( \boldsymbol{G}_k \boldsymbol{q}^l_{k+1} - \boldsymbol{p}(\boldsymbol{q}^l_{k+1}) \right), \quad l = 0, 1, 2, \ldots, \tag{25}$$

until the norm of the residual $\boldsymbol{g}(\boldsymbol{q}_{k+1}^l)$ is sufficiently small. Here the central function $\boldsymbol{p} : \mathbb{R}^{3s} \rightarrow \mathbb{R}^n$ is given by

$$p_i(\boldsymbol{q}) = \sqrt{2d_i^2 - \|\boldsymbol{q}_{a_i} - \boldsymbol{q}_{b_i}\|_2^2} \tag{26}$$

and the next state vectors are computed as follows

$$\boldsymbol{q}_{k+1} = \boldsymbol{q}_{k+1}^{l_{\max}} \tag{27}$$

$$\boldsymbol{v}_{k+\frac{1}{2}} = \frac{\boldsymbol{q}_{k+1} - \boldsymbol{q}_k}{h}. \tag{28}$$

where $l_{\max}$ is the final value of the index $l$. We are still investigating the deep mathematical reasons behind the fact that the LINCS sequence $\{\boldsymbol{q}_{k+1}^l\})_{l=0}^{\infty}$ converges to a solution of the constraint equation $\boldsymbol{g}(\boldsymbol{q}) = \boldsymbol{0}$, and how this property was formally established in the papers defining LINCS and P-LINCS[28,29]. However, in our numerical experiments, we have observed that the 2-norm of the residual $\boldsymbol{g}(\boldsymbol{q}_{k+1}^l)$ decays linearly until we reach the limits of floating point arithmetic and additional iterations are pointless. The LINCS algorithm hinges on our ability to apply the operator $\boldsymbol{T}_n$. Specifically, it is necessary to solve linear systems of the form

$$\left(\boldsymbol{G}_k \boldsymbol{M}^{-1} \boldsymbol{G}_k^T\right) \boldsymbol{x} = \boldsymbol{y} \tag{29}$$

where the right-hand side vector $\boldsymbol{y}$ and the unknown vector $\boldsymbol{x}$ are both elements of $\mathbb{R}^s$. Since the inception of the algorithm, the solution $\boldsymbol{x}$ has been approximated using a truncated Neumann series. Specifically, the coefficient matrix $\boldsymbol{G}_n \boldsymbol{M}^{-1} \boldsymbol{G}_n^T$ is subjected to a diagonal scaling, i.e.,

$$\boldsymbol{G}_k \boldsymbol{M}^{-1} \boldsymbol{G}_k^T = \boldsymbol{S}^{-1} \left(\boldsymbol{S} \boldsymbol{G}_k \boldsymbol{M}^{-1} \boldsymbol{G}_k^T \boldsymbol{S}\right) \boldsymbol{S}^{-1} \tag{30}$$

where $\boldsymbol{S} \in \mathbb{R}^{s \times s}$ is a diagonal matrix such the diagonal entries of the matrix $\boldsymbol{S}\boldsymbol{G}_n \boldsymbol{M} \boldsymbol{G}_k^T \boldsymbol{S}$ are all equal to one. This matrix is then viewed as a perturbation of the identity matrix $I_s$, i.e.,

$$(\boldsymbol{S} \boldsymbol{G}_k \boldsymbol{M}^{-1} \boldsymbol{G}_k^T \boldsymbol{S}) = \boldsymbol{I}_s - \boldsymbol{A}_k \tag{31}$$

and the action of the inverse of $\boldsymbol{S}\boldsymbol{G}_k\boldsymbol{M}^{-1}\boldsymbol{G}_k^T\boldsymbol{S}$ is approximated using a truncated Neumann series, i.e.,

$$\begin{aligned}\boldsymbol{x} = (\boldsymbol{G}_k\boldsymbol{M}^{-1}\boldsymbol{G}_k^T)^{-1}\boldsymbol{y} = \boldsymbol{S}(\boldsymbol{S}\boldsymbol{G}_k\boldsymbol{M}^{-1}\boldsymbol{G}_k^T\boldsymbol{S})^{-1}\boldsymbol{S}\boldsymbol{y} \\ = \boldsymbol{S}(\boldsymbol{I}_s - \boldsymbol{A}_n)^{-1}\boldsymbol{S}\boldsymbol{y} \approx \boldsymbol{S}\left(\sum_{j=0}^{I-1}\boldsymbol{A}_k^j\right)\boldsymbol{S}\boldsymbol{y}. \quad (32)\end{aligned}$$

for a modest value of the positive integer $I$ with is typically chosen as 4 or 8. The parameter $I$ is known as `lincs-order` in Gromacs. It is important to appreciate the fact that while there are known molecules for which this series does not converge —see the original paper[28]—, there is ample experience to suggest that the approximation works well in practice.

The P-LINCS algorithm is a straightforward parallelization of the LINCS algorithm and hinges on the fact that it is relatively easy to parallelize the matrix-vector multiplications needed to evaluate the right-hand side of equation (32).

# 3. Extended Description of ILVES-M and ILVES-F

In this section, we provide a detailed description of the ILVES-M and ILVES-F algorithms as well as their parallel implementations which we have integrated into Gromacs. This software was used to analyze the performance and accuracy of the algorithms presented in this study. While the implementation is fine-tuned for Gromacs, it can be straightforwardly migrated to any MD packages, such as Namd[35,36], Amber[37], Charmm[38], Lammps or Desmond.

ILVES-M solves the same system of equations (8) as SHAKE, but uses a quasi-Newton method to solve the constraint equations. If ILVES-M and SHAKE were all executed using exact arithmetic and if the constraint equations were solved exactly, then these algorithms would return the same results. Why is this? If we assume that the Jacobian of the constraint function is of full rank, then the Inverse Function Theorem implies that the constraint equations have a unique solution when the time step is sufficiently small. It follows inductively

that the ILVES-M and SHAKE algorithms must produce identical output.

ILVES-F also solves the same system of equations (8) as SHAKE, but it uses a quasi-Newton method to solve the constraint equations. The central matrix $\boldsymbol{A}\big(\boldsymbol{\phi}_k(\boldsymbol{\lambda}_{k,l}), \boldsymbol{q}_k^0\big)$ is a function of $l$, but it can be approximated by a constant matrix, i.e.,

$$\boldsymbol{A}\big(\boldsymbol{\phi}_k(\boldsymbol{\lambda}_{k,l}), \boldsymbol{q}_k^0\big) \approx \boldsymbol{A}\big(\boldsymbol{q}_k^0, \boldsymbol{q}_k^0\big) \tag{33}$$

simply because $\boldsymbol{\phi}_k(\boldsymbol{\lambda}) = \boldsymbol{q}_k + \mathcal{O}(h)$. It is therefore natural to replace Newton's method with the quasi-Newton method

$$\boldsymbol{A}\big(\boldsymbol{q}_k^0, \boldsymbol{q}_k^0\big)\boldsymbol{z}_{k,l} = \boldsymbol{g}\big(\boldsymbol{\phi}_k(\boldsymbol{\lambda}_{k,l})\big)\,, \tag{34}$$

$$\boldsymbol{\lambda}_{k,l+1} = \boldsymbol{\lambda}_{k,l} - \boldsymbol{z}_{k,l} \tag{35}$$

This approach is standard within the field of numerical analysis and different names such as the "simplified Newton's method"[39] or the "chord method"[40] are used. It is the foundation of the Constant Constraint Matrix approach used by Ref.[8] in the context of molecular dynamics. As described in this section, this approach enables ILVES-F to outperform ILVES-M.

ILVES-M and ILVES-F leverage three levels of parallelism: distributed memory parallelism, shared memory parallelism, and vectorization. To exploit distributed memory parallelism, our algorithms use a natural extension of the Overlapping Partitioning Method (OPM)[41] from banded to general sparse systems. In distributed-memory simulations with GROMACS, each domain also stores information about neighboring atoms, i.e., those within a distance of up to $I$ edges from any atom local to the domain, where $I$ is a user-defined parameter (`lincs-order` in the case of P-LINCS). Using both the local and neighboring atoms in each domain, a local approximation of the matrix-vector product $\mathbf{z} = \mathbf{A}^{-1}\mathbf{g}$ is computed. This approximation is computed in each domain using a multi-thread implementation of Gaussian elimination based on the Schur method[42]. As detailed in the paragraphs that follow, this approach partitions the linear system, distributing segments across the domain's threads for

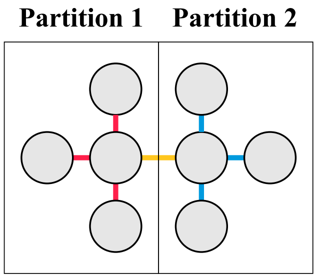


(a) Partitioning of graph

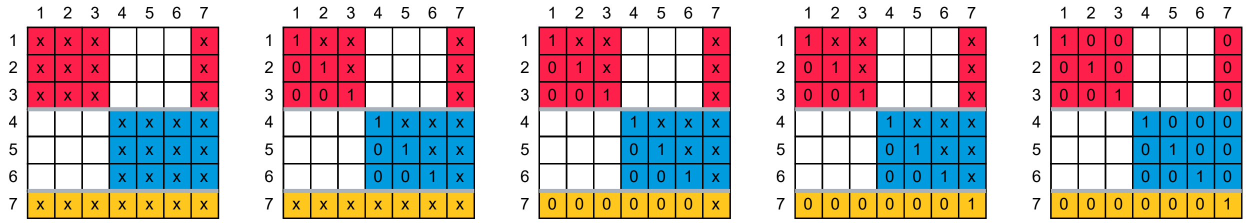

(b) Initial $A$ (c) Step 1 (d) Step 2 (e) Step 3 (f) Step 4

Figure S1: A possible partitioning of a simple graph (a), the corresponding coordinate matrix ($\boldsymbol{A}$) of the linear system of equations ($\boldsymbol{A}\big(\boldsymbol{\phi}_k(\boldsymbol{\lambda}_{k,l}), \boldsymbol{q}_k^0\big)\boldsymbol{z}_{k,l} = \boldsymbol{g}\big(\boldsymbol{\phi}_k(\boldsymbol{\lambda}_{k,l})\big)$) (b), and the steps to solve the linear system in parallel through Gauss-Jordan elimination and the Schur complement method (c-f). An analogous approach applies to solving the system with LU/LDLT decompositions and backward/forward substitution. Colors denote partitions, and each 'x' character represents a non-zero entry in the linear system. The red rows (1–3) and blue rows (4–6) represent private partitions for threads 1 and 2, respectively, while the yellow row (7) is the shared partition. To solve the system in parallel, each thread begins by zeroing the subdiagonal part of its private partition and setting the diagonal entries to ones (c). Next, the threads add their contributions to the shared partition in mutual exclusion (d). The master thread then performs Gauss-Jordan elimination on the shared partition (e), modifying only the last diagonal entry in this example. Once the master thread completes this step, all threads proceed in parallel to zero out the upper diagonal elements in their private partitions (f).

parallel processing. An example of how a domain partitions a simple graph and the steps followed to solve its corresponding linear system is presented in Fig. S1.

It is important to note that, in distributed memory simulations, GROMACS frequently rebuilds the list of atoms assigned to each domain, a data structure known as the neighbor list. This list determines which atoms are considered local to each domain and which neighboring atoms must be included for calculations such as constraints. The frequency of these updates is controlled by the `nstlist` parameter, whose default value is 10 steps. Each time the neighbor list is recomputed, the constraint solver must be reinitialized to reflect possible displacements of atoms between domains. Since the neighbor list is frequently recomputed, the performance of the initialization has a significant impact on the overall algorithm performance. Hence, we designed and implemented an initialization of ILVES-M and ILVES-F, which minimizes the time spent in this stage.

The atoms and bonds handled by the constraint solver in each domain can be represented as an undirected graph $G = (V, E)$. The vertices $V$ represent the atoms connected with at least one constrained bond and the edges $E \subseteq V \times V$ represent the constrained bonds. Since the simulation can contain more than one molecule and it is common to constrain only a subset of the bonds; for example, bonds that connect a hydrogen bond (H-bonds), $G$ is usually a disconnected graph. From $G$ we can compute its line-graph $L(G)$ in which each vertex represents a constrained bond, and two constrained bonds are connected if they share one vertex in $G$. The adjacency matrix of the line-graph has the same sparsity pattern as the coordinate matrices of the linear systems solved by ILVES-M and ILVES-F.

The graph $L(G)$ can be partitioned such that all the edges of $L(G)$ that do not cross partitions comprise each thread's private partition, while edges that connect vertices of $L(G)$ which lie in different private partitions comprise the shared partition. The data in the private partitions can be processed simultaneously, as they are independent of one another. In contrast, the data in the shared partition must be processed separately, in mutual exclusion from the private partitions, to ensure correctness and avoid conflicts.

While both P-LINCS and ILVES-M/F employ a partitioning of $L(G)$ for parallel execution, their methodologies differ significantly. P-LINCS partitions $L(G)$ directly dividing its vertices into roughly equal sets, followed by computation to identify edges crossing partitions. On the other hand, ILVES-M/F tries to minimize the edges crossing partitions using a greedy algorithm to partition $G$ rather than $L(G)$. Graphs in MD exhibit very low connectivity and, additionally, GROMACS assigns close indexes to connected atoms. Our algorithm sequentially assigns vertices of $G$ to partition $p$ while the partition size remains below a target value plus an offset. The final vertex in partition $p$ is chosen based on the number of edges cut and the similarity of the partition size to the target size. This greedy algorithm runs in $\mathcal{O}(|E|)$ time, where $|E|$ is the number of edges in $G$, and produces partitions comparable to those generated by general but slower graph-partitioning libraries like METIS[43], often cutting a similar or smaller number of edges relative to the number of partitions. Our partitioning approach produces significantly fewer cuts than P-LINCS', which results in notably less serial work and less synchronization between threads in cases where zero-cut partitions are possible, such as simulations of several small molecules.

The pseudocode of our greedy approach for partitioning a graph into $k$ partitions is presented in Fig. S2. For the results discussed in this study, the parameter `maxDeviation` was set to 0.05, and `cutPenalty` was assigned a value of 10.

After computing the partitioning of $L(G)$ we use a modified parallel implementation of the Approximate Minimum Degree ordering algorithm (AMD)[44] to minimize the fillins in the part of the linear system corresponding to each partition (a *fillin* is a zero entry of the coordinate matrix which becomes non-zero when computing a factorization such as an LU or an LDLT factorization which is needed for Gaussian elimination). This step completes the initialization of the ILVES algorithm, which is then ready to apply the constraints in each step until reinitialization is required after GROMACS recomputes its neighbor list.

ILVES-M and ILVES-F enforce constraints at each step through three primary phases: i) constructing the relevant linear systems and calculating the maximum relative error, ii)

Figure S2: Greedy algorithm for partitioning a graph into $k$ pieces

**Require:** $k$: number of partitions, $neighs$: sorted adjacency list where $neighs[v]$ contains the list of neighboring vertices for vertex $v$.

**Ensure:** Array $ids$ with the partition ID of each vertex.

```
Initialize maxDeviation and cutPenalty as problem-specific constants
ids ← vector of size |neighs|, initialized to −1
v ← 0
for p ← 0 to k − 1 do
    targetPsize ← (|neighs| − v)/(k − p)
    maxPsize ← targetPsize × (1 + maxDeviation)
    bestVertex ← −1
    bestScore ← ∞
    psize ← 0
    ncuts ← 0
    while v < |neighs| and psize ≤ maxPsize do
        if ids[v] = p then
            ncuts ← ncuts − 1
        else
            ids[v] ← p
        end if
        psize ← psize + 1
        i ← BinarySearch(v, neighs[v])
        for neigh in neighs[v] from index i + 1 onward do
            if ids[neigh] < p then
                ids[neigh] ← p
                ncuts ← ncuts + 1
            end if
        end for
        score ← |psize − targetPsize| + ncuts × cutPenalty
        if score < bestScore then
            bestVertex ← v
            bestScore ← score
        end if
        v ← v + 1
    end while
    v ← bestVertex + 1
end for
for v ← v to |neighs| − 1 do
    ids[v] ← k − 1
end for
return ids
```

solving the linear systems, and iii) updating atomic positions. These phases are iteratively executed until the desired tolerance for the maximum relative error is reached.

During the first phase, each thread constructs the corresponding portion of the linear system for its private partition, while the master thread also handles the shared partition. In this phase, the private and shared partitions can be computed concurrently. The computation of the maximum relative error is overlapped with the construction of the linear system. In distributed-memory simulations, domains exchange their maximum relative errors after this step is complete. Note that for ILVES-F, the coordinate matrix of the linear system of equations to solve ($\boldsymbol{A}$) remains constant for all iterations and is thus computed just once per simulated time. This step is vectorized using the GROMACS SIMD intrinsics interface.

Next, the Lagrange multipliers $\boldsymbol{\lambda}$ are calculated by solving the linear system in parallel through LU/LDLT factorization for ILVES-M/F. The factorization is followed by forward and backward substitutions. ILVES-F requires a single LDLT factorization per step because $\boldsymbol{A}$ remains unchanged for all the iterations in a given simulated time. This process is detailed in Fig. S1.

Finally, the positions of the atoms are updated. Updates to the positions of atoms in private partitions are processed in mutual exclusion to updates of atoms in the shared partition. In distributed-memory simulations, the updated atomic positions are exchanged between domains, ensuring that each domain has the latest positions of its neighboring atoms.

# 4. Further Discussion on the Need to Solve the Constraint Equations Accurately

We have presented arguments in favor of an accurate solving of the constraint equations in the main paper and in a recent publication[34]. In this section we present further arguments, supported by numerical evidence, to reinforce this thesis.

First, let us note that if the constraint equations are solved exactly, then the SHAKE

algorithm is second-order accurate in the time step. In practice, it is impossible to solve the constraint equations exactly and we accept an approximation if the relative error is less than the tolerance Tol passed to the constraint solver. In this section, we shall explain why the only safe value of Tol is tiny and why we should run the constraint solver until stagnation. We shall present arguments that should appeal to both the numerical analyst and the experimental scientist who seeks to validate a model of physical reality.

It is important to distinguish between the exact numbers that are defined by the SHAKE algorithm and the numbers that are computed when the algorithm is executed using finite precision arithmetic and tolerance Tol $> 0$.

Table S1: Parameters defining a simple pendulum.

| Parameter | Symbol | Value |
|---|---|---|
| Mass | $m$ | $1\ kg$ |
| Length | $l$ | $1\ m$ |
| Gravitational const. | $g$ | $9.82\ m/s^2$ |
| Initial velocity | $v_0$ | $(1,0)\ m/s$ |
| Initial position | $r_0$ | $(0,0)$ |

We begin by demonstrating that if the tolerance parameter Tol is small enough, then the computed numbers behave in a manner that is indistinguishable from the numbers that are defined by the SHAKE algorithm. To this end, we shall study a simple pendulum. This is a standard test problem. The necessary files to execute the calculations and regenerate our figures from scratch can be found in the public GitHub repository

`https://github.com/spockcc/_PAPER_ILVES_pendulum`

We shall demonstrate that if the tolerance in solving is small enough, then the total energy is very nearly preserved and the error is $\mathcal{O}(h^2)$. Our pendulum is defined by Tab. S1.

The MATLAB function `shake_pendulum` is included in the GitHub mentioned above. It does a sequence of 3 simulations of the same pendulum using different values of the time step and generates Fig. S3. The exact parameters of these simulations are given by Tab. S2.

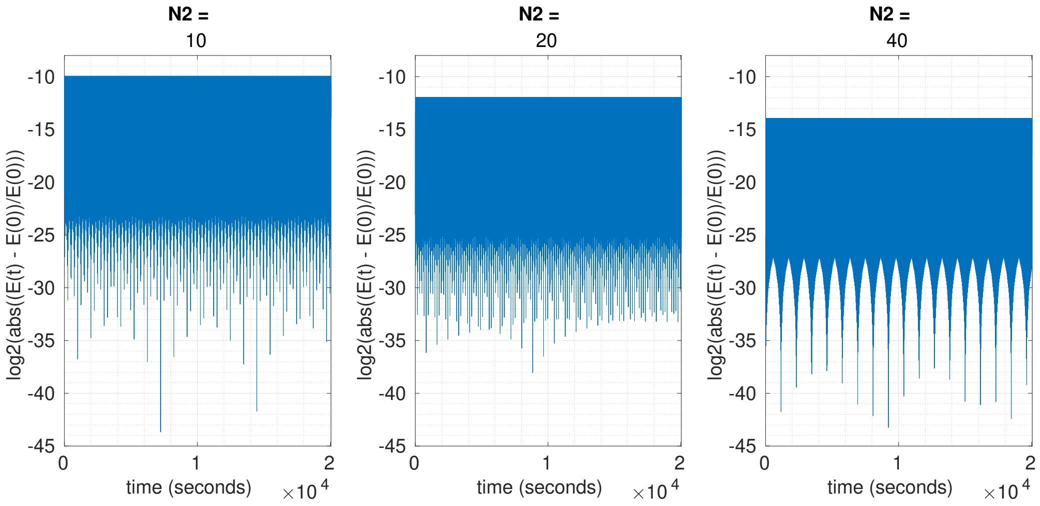


Figure S3: Total energy of a simple pendulum as a function of time for three different values of timestep.

Table S2: The parameters for the first set of simulations of the pendulum given by Tab. S1. The evolution of the total energy is shown in Fig. S3.

| Description | Name | Value |
|---|---|---|
| Number of periods | np | 10,000 |
| Number of recorded state vectors per period | nsp | 10 |
| Number of recorded state vectors | N1 | $\texttt{N1} = \texttt{np} \cdot \texttt{nsp}$ |
| Number of steps between recorded state vectors | N2 | $\{10, 20, 40\}$ |
| Tolerance passed to constraint solver | Tol | $10^{-12}$ |

We note that the simulations do $\texttt{nsp} \cdot \texttt{N2} \in \{100, 200, 400\}$ steps per period, which is far above the rule of thumb that suggests at least 5 steps per period. In Fig. S3 we see that the relative error of the total energy, i.e.,

$$R(t) = \frac{E(t) - E(0)}{E(0)} \tag{36}$$

is bounded as a function of the time $t$ and that the maximum value of the absolute value of relative error is approximately $\{2^{-10}, 2^{-12}, 2^{-14}\}$ for $\texttt{N2} = \{10, 20, 40\}$. This is experimental evidence in support of the hypothesis that the maximum relative error is $\mathcal{O}(h^2)$. This is the behavior that we would observe if the algorithm was executed using exact arithmetic and the constraint equations were solved exactly.

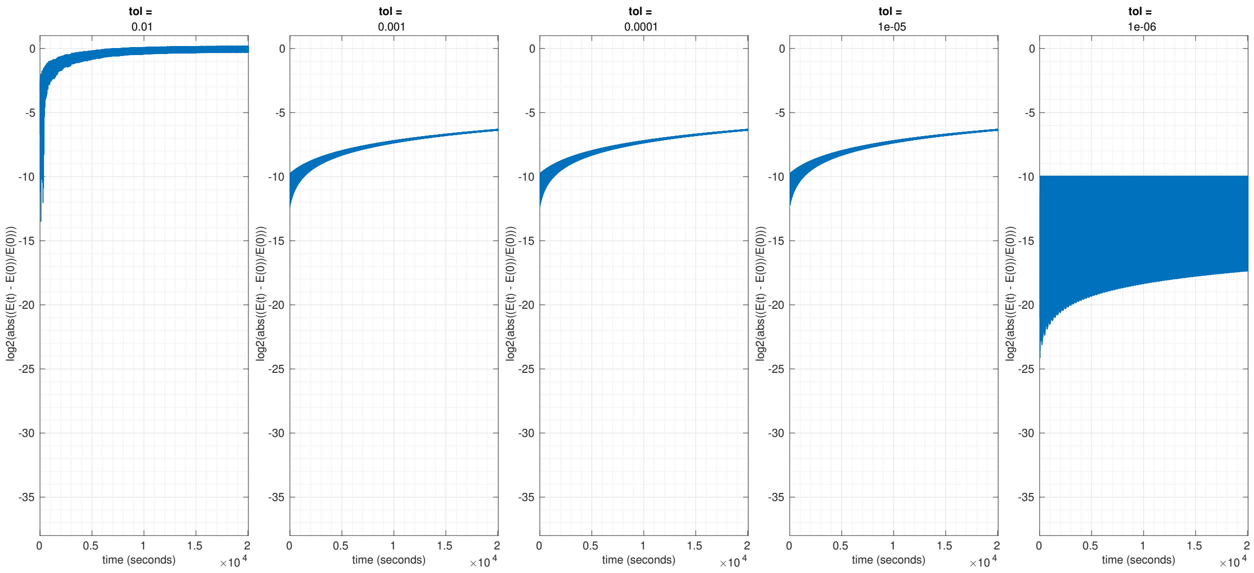


Figure S4: The evolution of the total energy of a pendulum over time for different values of the tolerance Tol passed to the constraint solver.

The MATLAB function `shake_pendulum` does a second sequence of 10 simulations of the same pendulum using different values of the tolerance Tol. The exact parameters are given by Tab. S3. The constraint solver is a quasi-Newton's method, specifically the simplified Newton's method that uses a fixed value of the Jacobian for each step. The linear convergence of this method ensures that the constraints are solved with a relative error that is essentially

Table S3: The parameters for the second set of simulations of a pendulum given by Tab. S1. The evolution of the total energy is displayed in Fig. S4.

| Description | Name | Value |
|---|---|---|
| Number of periods | `np` | 10,000 |
| Number of recorded state vectors per period | `nsp` | 10 |
| Number of recorded state vectors | `N1` | $\texttt{N1} = \texttt{np} \cdot \texttt{nsp}$ |
| Number of steps between recorded state vectors | `N2` | 10 |
| Tolerance passed to constraint solver | Tol | $\{10^{-2}, 10^{-3}, \dots, 10^{-6}\}$ |
| Constraint solver | `method` | `quasi` |

equal to the tolerance passed to the solver. Fig. S4 shows how the relative error $R(t)$ of the energy evolves over time for different values of the tolerance Tol. In particular, we see that the relative error is a strictly increasing function of $t$ for Tol$\in \{10^{-2}, 10^{-3}, 10^{-4}, 10^{-5}\}$. For these values of the tolerance Tol, we cannot claim that the simulation corresponds to an isolated system because the fundamental principle of the conservation of energy is clearly violated. However, for $\tau = 10^{-6}$, the maximum relative error is bounded as a function of $t$. Again, this is exactly the behavior we would observe if the algorithm were executed in exact arithmetic and the constraint equations were solved exactly.

These two numerical experiments show that if the tolerance parameter Tol is sufficiently small, then the total energy of the pendulum is very nearly preserved, and the computed numbers behave in a manner that is consistent with a method that is second-order accurate in the time step. If the tolerance is not sufficiently small, then the absolute value of the relative error of the energy is an increasing function of time.

In the absence of any deep understanding of the relationship between any system, the time step, the tolerance, and the evolution of the relative error of the energy, the only safe policy is to run the constraint solver until it stagnates due to the limitation of finite precision arithmetic. Naturally, this process is not practical unless the constraint solver converges rapidly.

The MATLAB function `shake_pendulum` also conducts a third sequence of numerical experiments. Here, the focus is on the sign of the relative error rather than on its absolute

Table S4: The parameters of a third set of simulations of the pendulum given by Tab. S1. The results are shown in Fig. S5.

| Parameter | Name | Value (s) |
|---|---|---|
| Number of periods | `np` | 10,000 |
| Number of recorded state vectors per period | `nsp` | 10 |
| Number of recorded state vectors | `N1` | `N1` $=$ `np` $\cdot$ `nsp` |
| Number of steps between recorded state vectors | `N2` | 10 |
| Tolerance passed to constraint solver | Tol | $10^{-4}$ |
| Constraint solver | `method` | {`newton`,`quasi`} |

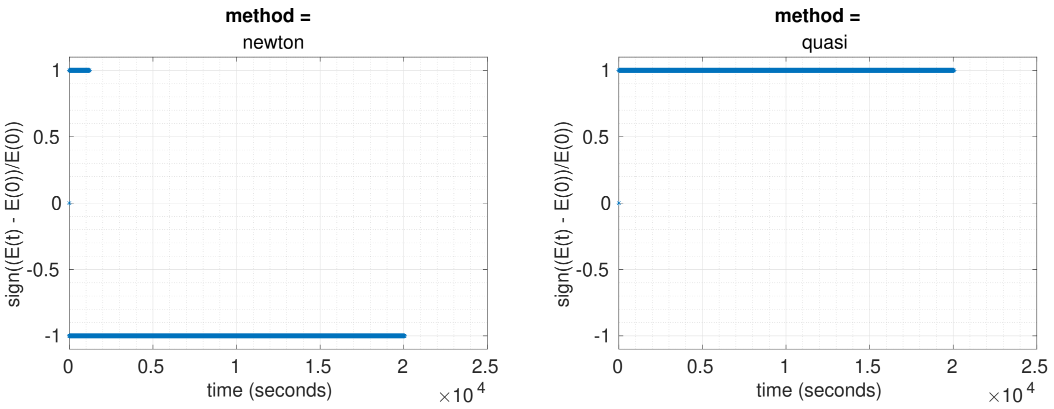


Figure S5: Sign of the relative error of the total energy for pendulum for two different constraint solvers as a function of the time: Newton's method (left) and the simplified Newton's method (right).

value. The two experiments reveal that the sign of the relative error can depend on the choice of the constraint solver. The exact parameters of these simulations are given by Tab. S4.

Fig. S5 shows the evolution of the sign of relative error of the energy. The scientist who uses Newton's method to probe the inner mysteries of the pendulum will (almost certainly) conclude that the system loses energy over time, whereas the scientist who uses the simplified Newton's method will conclude that the system gains energy over time. We find it intolerably dangerous that the physical conclusions drawn from a numerical experiment might depend on the algorithm used to solve the central equations.

We shall now argue further in favor of solving the constraint equations as accurately as the hardware allows. To this end, we ask the reader to consider a fundamental question: What is the purpose of an MD simulation? In general, we seek to obtain numbers that cannot be measured in the laboratory either because we lack the equipment or because the procedure is too expensive. However, before these experiments can be conducted it is necessary to validate our model and determine if it accurately captures the critical aspects of physical reality. In general, we do not care for the solution of a specific initial value problem for the system of differential algebraic equations (DAEs); rather we seek the value of a physical quantity that can be computed in terms of several such trajectories. Let $T$ denote the value that would be obtained if we could solve the relevant systems of DAEs exactly. Let $A_h$ be the value that would be obtained if we could solve the corresponding discrete-time equations exactly and let $\hat{A}_h$ denote the value of $A_h$ that is returned by the computer. It is important to recognize that the three numbers $T$, $A_h$, and $\hat{A}_h$ are almost certainly different. The discretization error $T - A_h$ is not zero because we have replaced the system of DAEs with a set of discrete equations. Moreover, the computational error $A_h - \hat{A}_h$ is not zero because we cannot solve the constraint equations exactly (truncation error) and because we are forced to use finite precision arithmetic (rounding error). Why should we care about these errors? We validate our model by comparing $T$ against the physical reality $P$. We cannot compute $T$ directly, and a small value of the observed error $P - \hat{A}_h$ does not necessarily imply that the modeling error

$P - T$ is small. Why is that? This issue can be understood using the elementary identity

$$\underbrace{(P-T)}_{\text{modeling error}} = \underbrace{(P-\hat{A}_h)}_{\text{observed error}} - \underbrace{(A_h-\hat{A}_h)}_{\text{computational error}} - \underbrace{(T-A_h)}_{\text{discretization error}} . \tag{37}$$

From this identity, we conclude that $T$ is a good approximation of $P$ if the computational error and the discretization error are both negligible compared with a small value of the observed error $P - \hat{A}_h$. It is, therefore, critical to control the size of the computational error and the discretization error. Frequently, but not universally, it is possible to use Richardson extrapolation[45,46] to estimate the discretization error $T - A_h$ by repeating the same simulation using different values of the time step $h$. In particular, Richardson extrapolation cannot be applied unless the force fields are sufficiently smooth[46]. Moreover, Richardson's error estimate is not reliable unless the computational error $A_h - \hat{A}_h$ is sufficiently small[46]. The size of the computational error $A_h - \hat{A}_h$ is controlled by the size of the unit roundoff $u$ and the tolerance Tol that is passed to the constraint solver. In the absence of any information about the size of the discretization error $T - A_h$, the safest course of action is, therefore, to use the smallest practical value of the unit roundoff $u$ and the tolerance Tol. This choice minimizes the computational error and maximizes the chance that the computational error is so small that we can reliably estimate the discretization error $T - A_h$. Therefore, it is prudent to favor double precision over single precision and tiny values of Tol over larger values of Tol. In ILVES we measure the relative constraint violation and iterate until

$$\frac{1}{2}\frac{\|\boldsymbol{q}_{a_i} - \boldsymbol{q}_{b_i}\|_2^2 - d_i^2}{d_i^2} \leq \text{Tol}$$

for all bonds or the maximum number of iterations is reached. If the relative constraint violation is small, then

$$\frac{1}{2}\frac{\|\boldsymbol{q}_{a_i} - \boldsymbol{q}_{b_i}\|_2^2 - d_i^2}{d_i^2} = \frac{1}{2}\frac{(\|\boldsymbol{q}_{a_i} - \boldsymbol{q}_{b_i}\|_2 - d_i)(\|\boldsymbol{q}_{a_i} - \boldsymbol{q}_{b_i}\|_2 + d_i)}{d_i^2} \approx \frac{\|\boldsymbol{q}_{a_i} - \boldsymbol{q}_{b_i}\|_2 - d_i}{d_i}. \tag{38}$$

On a machine with unit roundoff $u$ we cannot hope to reliably reduce the right-hand side below $u$. Therefore, the smallest value of Tol that should be considered is literally the unit roundoff $\text{Tol} = u$.

In conclusion, we can say the following: If we do not solve the constraint equations accurately, then we cannot claim that our calculations are $m$-th order accurate in the time step, we cannot assume that the fundamental principle of conversation of energy is respected, and we cannot reliably estimate the discretization error and perform the validation needed to develop faith in our model of the physical reality. This strong reason supports solving the constraints as accurately as possible.

# 5. Experimental Setup

Table S5: Main features of the computing nodes used to execute the simulations.

| | |
|---|---|
| Processor | 2 × Intel Xeon Platinum 8480+ |
| Cores | 2 × 56 |
| Frequency | 2 GHz |
| L1i cache (I + D) | 32 KB + 48 KB (per core) |
| L2 cache | 2 MB (per core) |
| LLC | 105 MB (shared) |
| Main Memory | 256 GB DDR5 (16 × 16 GB 4800 MHz DIMMs) |
| Interconnection | ConnectX-7 NDR200 InfiniBand (100 Gb/s per node) |

The simulations presented in this text were performed on a multi-node cluster. Each node is equipped with two Intel Xeon Platinum 8480+ chips, each with 56 cores. The main features of the nodes are summarized in Tab. S5 (see[47] for a presentation of high-performance computing). We used Gromacs 2021.0 compiled with GCC-10.1.0 for the numerical experiments.

Table S6: Molecular systems simulated in this article. In the Force Field column, C22+CMAP is Charmm22+CMAP, C36m is Charmm36m and C36 is Charmm36. Although the ff19SB force field defines residue-specific CMAP corrections, the protein topology of the DNA-protein system contains a generic CMAP matrix (taken from the `frcmod.ff19SB_XXX` internal file of AmberTools24) for all residues, due to the use of the `amb2gro_top_gro.py` script for conversion from Amber to Gromacs format, which ensures compatibility with versions prior to 2025.

| **System** | **# Molec. entities (PDB ID)** | **# Mol.** | **# Resid./ Atoms** | **# Constr. all-bonds/ H-bonds** | **Force Field/ Water Model** |
|---|---|---|---|---|---|
| Solvated barnase[48] | barnase (1A2P) | 1 | 108/1,705 | 1,721/836 | C22+CMAP[49] |
| | Water | 62,848 | 188,544 | — | Tip3p[50] |
| | NA$^+$, CI$^-$ | 4/11 | 15 | — | C22+CMAP[49] |
| Solvated nuclease[51] | nuclease (2SNS) | 1 | 149/2,398 | 2,459/1,224 | C22+CMAP[49] |
| | Water | 115,602 | 346,806 | — | Tip3p[50] |
| | CI$^-$ | 10 | 10 | — | C22+CMAP[49] |
| Solvated COVID protease[52] | protease (5R7Y) | 1 | 304/4,645 | 4,697/2,297 | C22+CMAP[49] |
| | Water | 61,958 | 185,874 | — | Tip3p[50] |
| | NA$^+$, CI$^-$ | 8/4 | 12 | — | C22+CMAP[49] |
| Pure benzene[53] | benzene | 2000 | 24,000 | 24,000/12,000 | Gromos54a7[54] |
| Solvated DNA-proteins[55] | histones (3LEL) | 16 | 1,540/25,257 | 25,933/13,078 | ff19SB[56] |
| | DNA | 4 | 588/18,712 | 20,178/6,670 | OL21[57] |
| | Water | 83,872 | 251,616 | — | OPC4[58] |
| | NA$^+$ | 428 | 428 | — | Ions[59,60] |
| Aquaporin 1 embedded in lipid bilayer[61] | Aqp1 (1J4N) | 4 | 996/15,072 | 15,480/7,755 | C36m[62] |
| | MMPC | 1,129 | 133,222 | 131,837/81,193 | C36[63] |
| | Water | 80,598 | 241,794 | — | Tip3p[50] |
| | NA$^+$, CI$^-$ | 223/235 | 458 | — | C36[63] |

# 6. Simulations

We have conducted simulations on a diverse set of molecular systems, including three solvated proteins, a solvated protein-DNA complex, an organic solvent, and a membrane protein embedded in a lipid bilayer surrounded by water. Tab. S6 summarizes the key characteristics of the systems, while Fig. S6 provides a visual representation. Biological macromolecules, such as DNA and proteins, have long been of interest for medical and biotechnological purposes. Benzene, as a representation of small molecules, has important chemical and industrial applications[64]. Proteins embedded in a lipid bilayer include channels, transporters, and signal receptors, among others, which play a vital role in cell biology and medical research[65]. This diversity of molecules allowed us to evaluate the performance of our ILVES algorithms across a wide range of molecular structures.

Below we outline the procedures on which the simulations are based. All the systems, except the protein (human aquaporin-1) embedded in a lipid bilayer, were prepared through a standard protocol involving solvation, minimization, heating, and equilibration. The most important parameters used in the preparation and also in the production phase of the simulations are listed in Tab. S7. The membrane protein system was prepared using the membrane-builder wizard from the `CHARMM-GUI` online tool[66–69]. We used different force fields for the simulations (Tab. S6), and explicit Tip3p water molecules[70] were used to solvate the macromolecular systems, except for the protein-DNA complex, which was solvated with the OPC4 water model[71]. The ionizable residues of proteins were protonated at pH 7 (Gromacs default), except for the case of barnase, which was set to pH 4.1 because the experimental thermodynamic observables used as a control were obtained under this condition. Chloride and/or sodium ions were added to neutralize the systems or to achieve the desired ionic strength. A cubic box was used for the membrane system, while a truncated dodecahedral box was used for the rest of the systems. The Verlet cutoff scheme[30,72] was applied for van der Waals interactions, and the Particle Mesh Ewald (PME) method[73] was used to account for electrostatics. Both methods were used with a radius cutoff of 1.0 nm (0.9 nm for the

Table S7: Relevant input parameters employed in the stages of the simulations.

| Step | General Settings | Thermodynamic Ensemble (baths) | Physical Conditions | Time step / Simulated Time |
|---|---|---|---|---|
| Min. | Integrator: steepest descent<br>Neighboring searching: grid<br>rcoul (PME): 1.0 $(0.9)^b$ nm<br>rvdw (cut-off): 1.0 $(0.9)^b$ nm<br>PBC: xyz<br>Constr: none | — | Emtol: 1 kJ/mol<br>Temp: 0 K<br>Press: 0 atm | max 20K steps |
| Heating | Integrator: md<br>Neighboring searching: grid<br>rcoul (PME): 1.0 $(0.9)^b$ nm<br>rvdw (cut-off): 1.0 $(0.9)^b$ nm<br>vdw-mod: potential-shift-verlet<br>PBC: xyz<br>Constr: all-bonds | Termost: Berendsen<br>$\tau_T = 0.1$ ps | Temp: Ini-T +<br>ramp (n $\times$ 50 K)<br>Press: 0 atm | 1 fs / n $\times$ 50 ps |
| Equil. 1 | Same as Heating | NVT<br>Termost: v-rescale<br>$\tau_T = 0.1$ ps | Temp: Final-T<br>Press: 0 atm | 1 fs / 100 ps |
| Equil. 2 | Same as Heating | NPT<br>Termost: v-rescale<br>$\tau_T = 0.1$ ps<br>Barost: Berendsen<br>$\tau_P = 2.0$ ps | Temp: Final-T<br>Press: 1 atm | 2 fs / 100 ps |
| Equil. 3 | Same as Heating | NPT<br>Termost: v-rescale<br>$\tau_T = 0.1$ ps<br>Barost: Parrinello-Rahman<br>$\tau_P = 2.0$ ps | Same as<br>Equil. 2 | 2 fs / 200 ps |
| Prod. | Same as Heating +<br>Constr: h-bonds,<br>all-bonds or h-angles | Same as Equil. 3 | Same as<br>Equil. 2 | 2 fs or 3.5 fs /<br>0.1 ns or 2 ns /<br>or 5.25 ns |

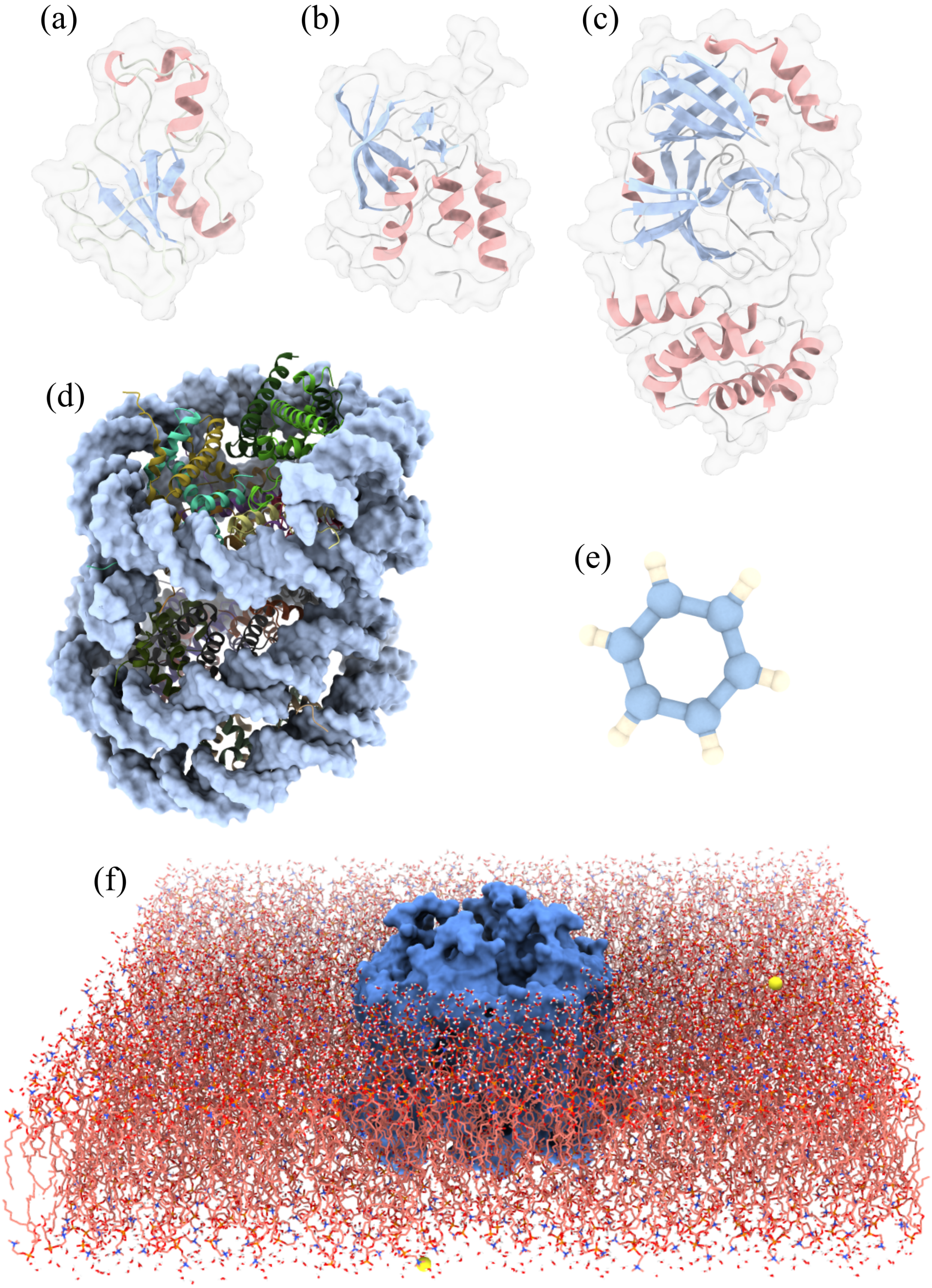


Figure S6: Representations of the simulated molecules, covering solvated proteins: (a) barnase (PDB 1A2P), (b) nuclease (PDB 2SNS) and (c) Covid-19 main protease (PDB 5R7Y); (d) a solvated protein-DNA complex (PDB 3LEL); (e) an organic solvent (benzene); and (f) a tetrameric protein embedded in a lipid bilayer.

membrane protein), while all the preparation steps imposed periodic boundary conditions (PBC).

The minimization step was performed using the steepest descent algorithm[74], followed by an NVT gradual heating protocol, increasing temperature by 50 K every 50 ps with a 1 fs time step, until the target temperature was reached. The equilibration process began with a 100 ps NVT simulation with a 2 fs time step using the V-rescale thermostat[75] ($\tau_T = 0.1$ ps). During this step, position restraints were applied to all the heavy atoms (protein, DNA, lipids, and water). This was followed by a 100 ps simulation in the NPT ensemble using a 2 fs time step and the heavy atoms now unrestrained. V-rescale thermostat ($\tau_T = 0.1$ ps) and the Berendsen barostat[76] (p = 1 atm, $\tau_P = 2.0$ ps) were settled. Finally, the system underwent 200-ps NPT simulation with a 2 fs time step, employing the V-rescale thermostat ($\tau_T = 0.1$ ps) and the Parrinello-Rahman barostat[77] (p = 1 atm, $\tau_P = 2.0$ ps). Temperature corrections due to the thermostat were applied every 10 steps (GROMACS' `nsttcouple` default value). Input files from our simulations can be retrieved from the provided `GitHub` repository (`https://github.com/LorienLV/_PAPER_ILVES`).

The final equilibrated configuration served as the starting point for the production phase of the simulations performed in our reliability and performance studies.

# 7. Reliability Study

In a previous work[78,79], one of the authors presented an MD-based method that has proven accurate in reproducing protein folding thermodynamics using both SHAKE and P-LINCS. In this paper, we applied that approach to evaluate the reliability of our constraint solvers by calculating three thermodynamic observable quantities: namely, the change in enthalpy upon unfolding $\Delta H_{unf}$ (or upon folding, with opposite sign); the change in heat capacity upon the same event, $\Delta Cp_{unf}$; and the corresponding change in Gibbs free energy (i.e., the protein conformational stability), $\Delta G_{unf}$. The analysis was conducted by simulating two well-studied

proteins: barnase and nuclease (see Tab. S6), which have been similarly modeled in Refs.[78,79]. Tab. S7 includes the main parameters used in the preparation and production simulations carried out with these systems.

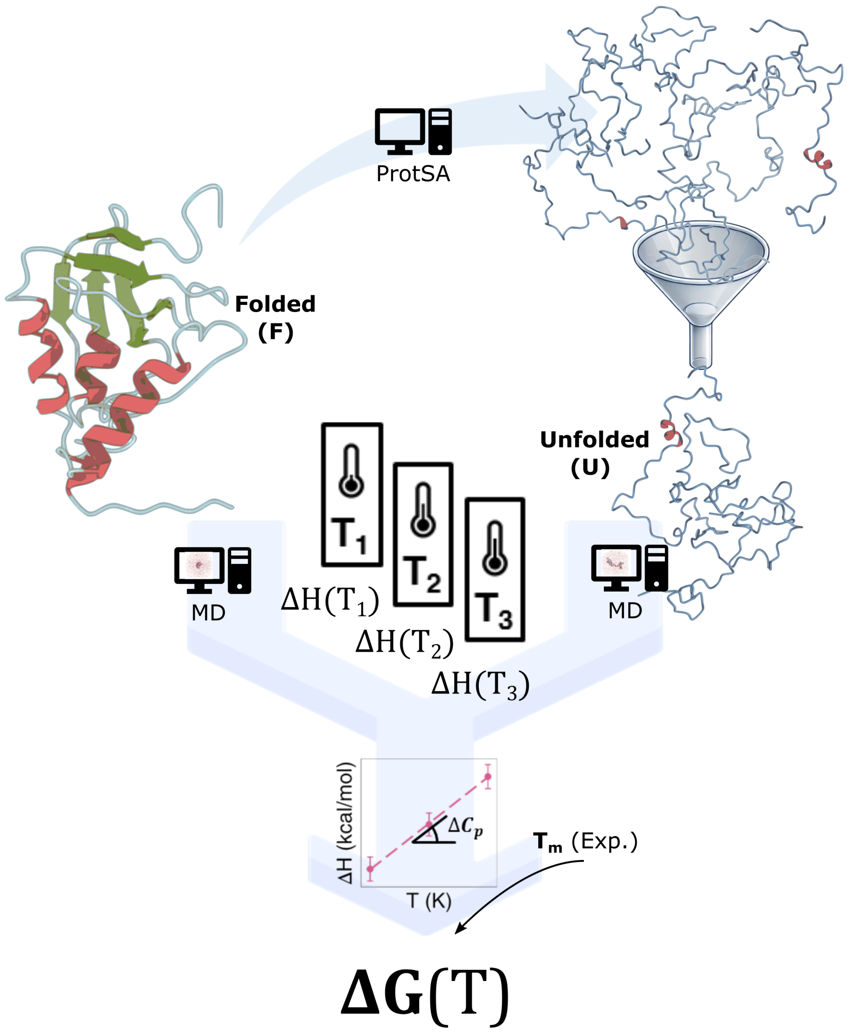


Figure S7: Workflow of the calculation of observable quantities.

Tab. S8 displays the results of our reliability study. The methodology of the study involves separately simulating—in the NPT ensemble—the folded and unfolded states of a solvated protein (see Fig. S7) under identical conditions (force field, type and number of water molecules, ions, pH, temperature, pressure, time step, simulation length, etc.). To achieve precise averaging of thermodynamic quantities, the method requires representative sampling of the conformational space of both states. We used 40 simulation replicas for the

Table S8: Experimentally measured and calculated (MD) thermodynamic quantities of unfolding ($\Delta H_{unf}$ and $\Delta Cp_{unf}$) for barnase ($T_m = 323.0 \pm 0.7$ at pH ~4.1[79]) and nuclease ($T_m = 326.9 \pm 0.4$ at pH ~7.0[79]). The listed Gibbs free energies ($\Delta G_{unf}$) are calculated through the Gibbs-Helmholtz equation using either the measured or MD thermodynamics. P-LINCS is configured with GROMACS default parameters, while MP-LINCS uses `lincs-order=4`. All of the simulations are performed with `constraints=h-bonds`.

| **Protein (Method)** | **Solver** | **Tol.** | **$\Delta H_{unf}$ at $T_m$ (kcal/mol)** | **$\Delta Cp_{unf}$ (kcal/mol · K)** | **$\Delta G_{unf}$ at 298 K (kcal/mol)** |
|---|---|---|---|---|---|
| Barnase (Exp.) | — | — | $118.7 \pm 4.9$ | $1.4 \pm 0.1$ | $7.8 \pm 1.6$ |
| Barnase (MD) | SHAKE | $10^{-4}$ | $113.9 \pm 3.1$ | $1.1 \pm 0.1$ | $7.8 \pm 1.1$ |
| | SHAKE | $10^{-10}$ | $115.2 \pm 3.1$ | $1.3 \pm 0.1$ | $7.6 \pm 1.4$ |
| | MP-LINCS | $10^{-4}$ | $116.9 \pm 3.1$ | $1.2 \pm 0.0$ | $7.9 \pm 1.2$ |
| | MP-LINCS | $10^{-10}$ | $118.6 \pm 3.1$ | $1.3 \pm 0.1$ | $7.9 \pm 1.3$ |
| | P-LINCS | — | $116.3 \pm 3.1$ | $1.2 \pm 0.0$ | $7.9 \pm 1.2$ |
| | ILVES-M | $10^{-4}$ | $113.6 \pm 3.1$ | $1.0 \pm 0.1$ | $7.8 \pm 1.2$ |
| | ILVES-M | $10^{-10}$ | $117.4 \pm 3.1$ | $1.1 \pm 0.2$ | $8.0 \pm 1.3$ |
| | ILVES-F | $10^{-4}$ | $116.4 \pm 3.1$ | $1.2 \pm 0.0$ | $7.8 \pm 1.2$ |
| | ILVES-F | $10^{-10}$ | $115.6 \pm 3.1$ | $1.0 \pm 0.1$ | $7.9 \pm 1.2$ |
| Nuclease (Exp.) | — | — | $82.1 \pm 4.7$ | $2.3 \pm 0.1$ | $4.2 \pm 1.6$ |
| Nuclease (MD) | SHAKE | $10^{-4}$ | $78.1 \pm 4.8$ | $2.0 \pm 0.1$ | $4.2 \pm 1.4$ |
| | SHAKE | $10^{-10}$ | $73.5 \pm 4.8$ | $1.9 \pm 0.1$ | $4.1 \pm 1.3$ |
| | MP-LINCS | $10^{-4}$ | $74.6 \pm 4.8$ | $1.8 \pm 0.1$ | $4.2 \pm 1.3$ |
| | MP-LINCS | $10^{-10}$ | $71.6 \pm 4.8$ | $1.7 \pm 0.2$ | $4.1 \pm 1.4$ |
| | P-LINCS | — | $70.8 \pm 4.8$ | $1.6 \pm 0.0$ | $4.2 \pm 1.1$ |
| | ILVES-M | $10^{-4}$ | $75.8 \pm 4.8$ | $2.0 \pm 0.1$ | $4.0 \pm 1.4$ |
| | ILVES-M | $10^{-10}$ | $74.6 \pm 4.8$ | $2.0 \pm 0.1$ | $4.0 \pm 1.4$ |
| | ILVES-F | $10^{-4}$ | $74.4 \pm 4.8$ | $2.0 \pm 0.0$ | $4.0 \pm 1.3$ |
| | ILVES-F | $10^{-10}$ | $73.1 \pm 4.8$ | $1.8 \pm 0.1$ | $4.1 \pm 1.3$ |

folded state and 100 replicas for the unfolded state, with a time step of 2 fs. The 100 protein structures representative of the unfolded state is a filtered sample of a larger set of completely unfolded conformations generated by the ProtSA server[80]. The average enthalpies obtained for the two states are subtracted to yield the enthalpy change upon unfolding, $\Delta H_{unf}$. From this calculation, the error is obtained as the sum of both errors: that corresponding to the folded ensemble and that of the unfolded ensemble. The calculation of $\Delta H_{unf}$ is performed at three temperatures (295 K, 315 K, and 335 K), allowing for the determination of the enthalpy change with temperature, which is typically linear. This linear relationship, whose slope corresponds to the change in heat capacity, $\Delta Cp_{unf}$, allows interpolating the enthalpy change at the mid-denaturation temperature ($T_m$, taken from the literature), which is the enthalpy change that is commonly reported in scientific articles. The reported error in $\Delta Cp_{unf}$ calculation is that obtained from the linear fitting. Once these two thermodynamic quantities are calculated ($\Delta H_{unf}$ at the $T_m$ and $\Delta Cp_{unf}$), the Gibbs free energy change can be obtained through the Gibbs-Helmholtz equation[81]:

$$\Delta G(T) = \Delta H_m \left(1 - \frac{T}{T_m}\right) - \Delta C_p \left[T_m - T + T \ln\left(\frac{T}{T_m}\right)\right]. \tag{39}$$

The reported error in $\Delta G(T)$ calculations (Tab. S8) is obtained by error propagation. In this assessment, we used the combination of force field and water model that has been shown to best reproduce experimental protein thermodynamics[78,79]: Charmm22+CMAP[49] and Tip3p[50].

The results displayed in Tab. S8 indicate that for ILVES and state-of-the-art solvers, the evaluated thermodynamics $\Delta H_{unf}(T_m)$ and $\Delta G_{unf}$(298K) fall within the error ranges defined by experimental measurements, for both proteins. In the case of $\Delta Cp_{unf}$, the MD-calculated values fall outside the experimental ranges in some of the setups assessed, but the differences are similar for all constraint solvers. This outcome supports the thesis that ILVES algorithms preserve system dynamics and do not introduce distortions larger than those due

to traditionally used constraint solvers.

# 8. Extended Performance Results

In this section, we complement the performance results presented in the main paper.

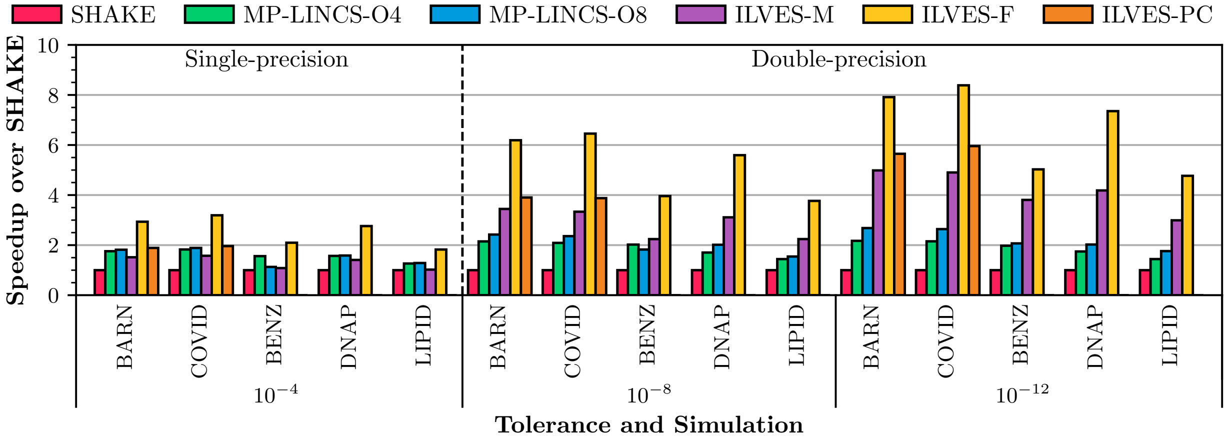


(a) all-bonds

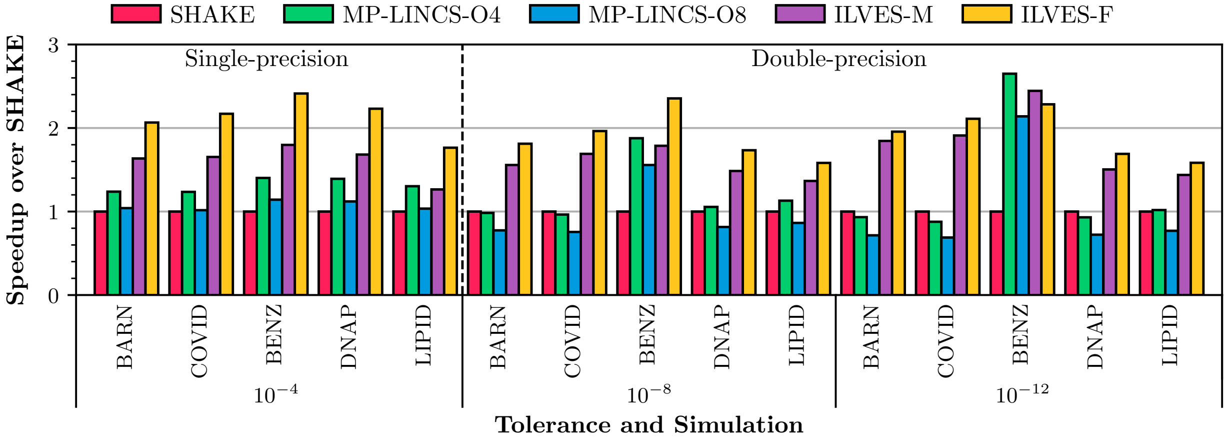


(b) H-bonds

Figure S8: Single-thread speedup over SHAKE of MP-LINCS (`lincs-order=4` and `lincs-order=8`), ILVES, ILVES-F and ILVES-PC.

Fig. S8 displays the *single-thread* speedup over SHAKE. This metric is defined as the ratio of the single-thread execution time of SHAKE to the single-thread execution time of the solver. While ILVES-PC is tailored to protein simulations, it delivers only modest speedups over ILVES-M, which incorporates optimizations absent in ILVES-PC, the most significant

being the vectorization of linear-system construction. When constraining all-bonds and using Tol $= 10^{-4}$, ILVES-M and ILVES-PC achieve speedups between $1\times$ and $1.8\times$ compared to SHAKE, similar to MP-LINCS. For the same tolerance, ILVES-F outperforms both, delivering speedups between $1.9\times$ and $3.2\times$ over SHAKE and between $1.5\times$ and $1.8\times$ over MP-LINCS. As the tolerance becomes more demanding, all ILVES versions show greater speedups, with a maximum of $8.4\times$ over SHAKE and $4.2\times$ over MP-LINCS. For `constraints=h-bonds`, MP-LINCS performs similarly to SHAKE in most simulations, while ILVES-M and ILVES-F show consistent speedups over both SHAKE and MP-LINCS across all tolerances, achieving maximum speedups of $2.4\times$ and $3\times$ over SHAKE and MP-LINCS, respectively. The only case in Fig. S8 where MP-LINCS outperforms ILVES-F is the BENZ simulation at the tightest tolerance. In it, each benzene molecule has only six constraints, and MP-LINCS achieves low errors in its projection step, sometimes avoiding any iteration of its correction step. Note that the benzene molecule with constraints on just H-bonds is a very exceptional case where every constraint is fully decoupled from the rest. This makes the matrix that must be inverted (in the involved linear systems of equations) diagonal because there exists no atom that belongs to two different constraints, which makes the linear system suitable for a faster analytical solution. Relevant research works recommend constraining both bond lengths and bond angles in benzene[25].

Fig. S9 illustrates the parallel scalability of the constraint algorithms. The scalability of a solver is defined as the ratio of the execution time of the solver using $N$ threads to the execution time of the solver using 1 thread. The parallel scalability of the ILVES algorithms is significantly better in the BENZ and DNAP simulations with `constraints=all-bonds`. In these simulations, the graph partitioning produced by ILVES-M results in substantially less serial work compared to MP-LINCS. In the case of BENZ, the partitioning eliminates serial work entirely. The strong scalability of the BENZ system may seem counter-intuitive given that benzene molecules are small. However, the system consists of 2,000 benzene molecules, leading to a substantial parallel workload. While in other simulations the scalability of the

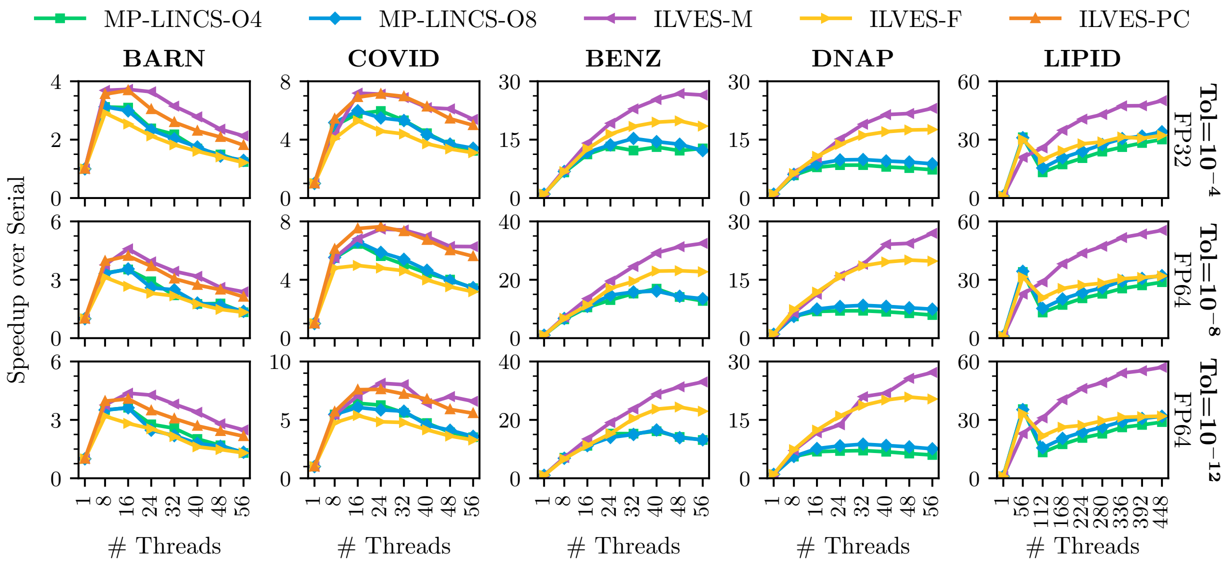


(a) All bonds

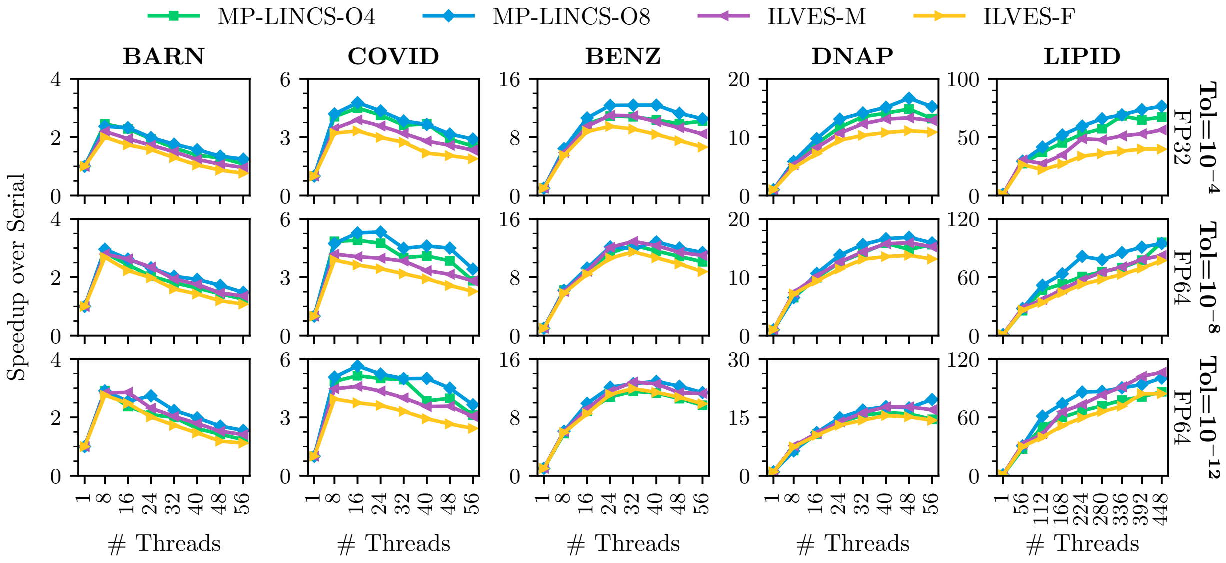


(b) H-bonds

Figure S9: Multi-thread speedup over serial execution of MP-LINCS (`lincs-order=4` and `lincs-order=8`), ILVES-M, ILVES-F and ILVES-PC. The BARN, COVID, BENZ, and DNAP simulations are executed using a single task in a single chip of a computing node. The LIPID simulation is executed using up to 8 tasks (up to 4 nodes and 8 chips). (a) Constraints imposed on all bonds; (b) On H-bonds.

ILVES algorithms and MP-LINCS is more similar, it is worth noting that ILVES delivers better performance than MP-LINCS in most cases. Consequently, for shorter execution times, the overhead of thread synchronization in ILVES becomes more pronounced, ultimately affecting its parallel scalability.

In most parallel simulations peak performance is not achieved using the maximum number of threads available. As more threads are added, synchronization overhead increases, and if zero-cut partitions are not possible, the amount of serial code executed also rises. When the parallel work becomes insufficient to offset the synchronization cost, the performance begins to decline. This behavior is particularly evident in the lipid bilayer simulation with `constraints=all-bonds`, where a performance drop occurs beyond 56 threads. This decline corresponds to the transition from a single task to multiple tasks, which increases synchronization and serial execution. In contrast, this drop is absent with `constraints=h-bonds` due to the lack of bonds between atoms in different tasks, thereby eliminating the need for inter-task communication.

Fig. S10 displays the percentage of the total simulation execution time spent in the constraint solver (excluding water). In our multi-threaded simulations, with `constraints=all-bonds`, SHAKE accounts for up to 92% of the total execution time, MP-LINCS up to 42%, and ILVES up to 16%. With `constraints=h-bonds`, SHAKE accounts for up to 60%, MP-LINCS up to 6%, and ILVES up to 5%.

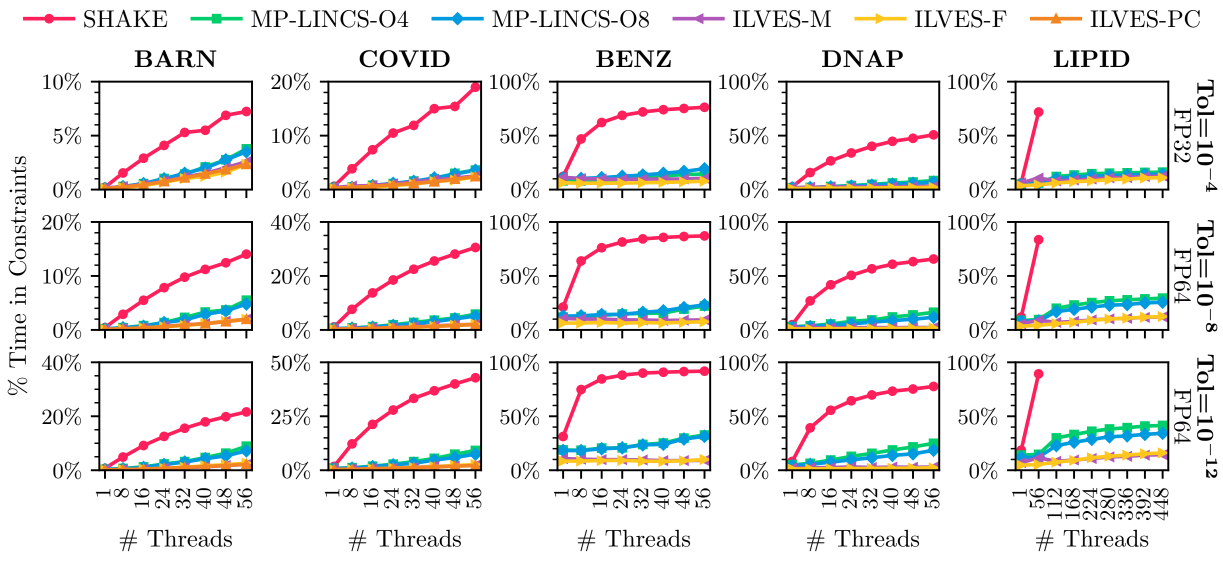


(a) All bonds

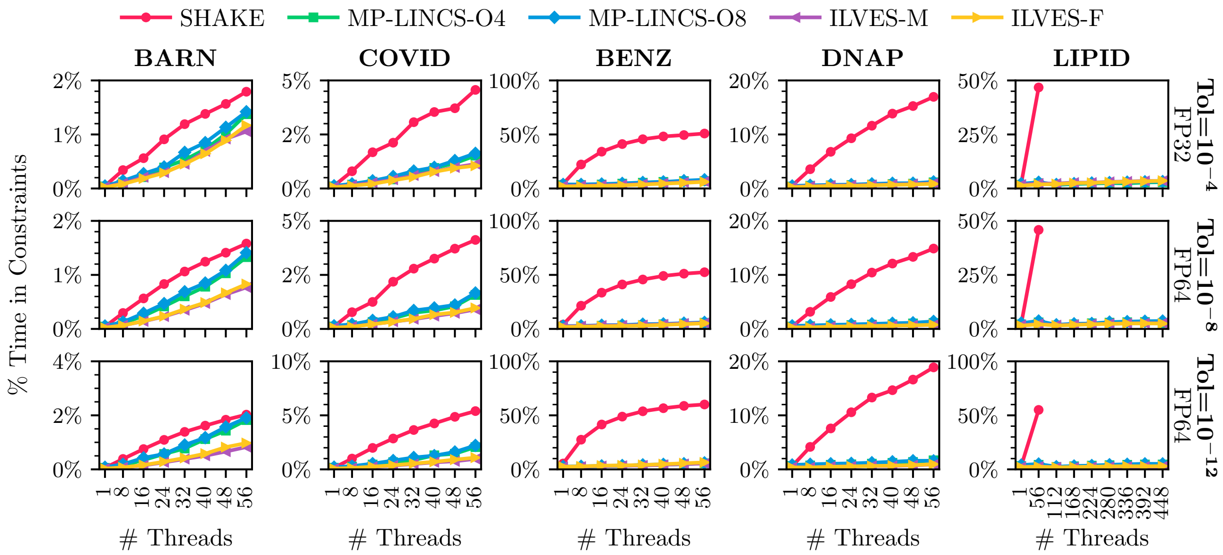


(b) H-bonds

Figure S10: Percentage of the total execution time of `Gromacs` spent in the constraint solver (ex water). (a) constraints imposed on all bonds; (b) On H-bonds.

# References


(1) Andersen, H. C. Rattle: A "velocity" version of the SHAKE algorithm for Molecular Dynamics calculations. *Journal of Computational Physics* **1983**, *52*, 24–34.

(2) Miyamoto, S.; Kollman, P. A. Settle: An analytical version of the SHAKE and RATTLE algorithm for rigid water models. *Journal of Computational Chemistry* **1992**, *13*, 952–962.

(3) Lambrakos, S. G.; Boris, J.; Oran, E.; Chandrasekhar, I.; Nagumo, M. A modified SHAKE algorithm for maintaining rigid bonds in molecular dynamics simulations of large molecules. *Journal of Computational Physics* **1989**, *85*, 473–486.

(4) Lee, S.-H.; Palmo, K.; Krimm, S. WIGGLE: A new constrained molecular dynamics algorithm in Cartesian coordinates. *Journal of Computational Physics* **2005**, *210*, 171–182.

(5) Gonnet, P. P-SHAKE: A quadratically convergent SHAKE in O(n2). *Journal of Computational Physics* **2007**, *220*, 740–750.

(6) Ciccotti, G.; Ryckaert, J.-P. Molecular dynamics simulation of rigid molecules. *Computer Physics Reports* **1986**, *4*, 346–392.

(7) Ryckaert, J.-P.; Arialdi, G.; Melchionna, S. Molecular dynamics of polymers with explicit but frozen hydrogens. *Molecular Physics* **2001**, *99*, 155–165.

(8) Eastman, P.; Pande, V. S. Constant Constraint Matrix Approximation: A robust, parallelizable constraint method for molecular simulations. *Journal of Chemical Theory and Computation* **2010**, *6*, 434–437.

(9) Krautler, V.; Van Gunsteren, W. F.; Hunenberger, P. H. A fast SHAKE Algorithm to Solve Distance Constraint Equations for Small Molecules in Molecular Dynamics Simulations. *Journal of Computational Chemistry* **2001**, *22*, 501–508.

(10) Christen, M.; van Gunsteren, W. F. An approximate but fast method to impose flexible distance constraints in molecular dynamics simulations. *The Journal of Chemical Physics* **2005**, *122*.

(11) Bailey, A. G.; Lowe, C. P.; Sutton, A. P. Efficient constraint dynamics using MILC SHAKE. *Journal of Computational Physics* **2008**, *227*, 8949–8959.

(12) Bailey, A. G.; Lowe, C. P. MILCH SHAKE: An efficient method for constraint dynamics applied to alkanes. *Journal of Computational Chemistry* **2009**, *30*, 2485–2493.

(13) Mazars, M. Holonomic constraints: an analytical result. *Journal of Physics A: Mathematical and Theoretical* **2007**, *40*, 1747–1755.

(14) Weinbach, Y.; Elber, R. Revisiting and parallelizing SHAKE. *Journal of Computational Physics* **2005**, *209*, 193–206.

(15) Elber, R.; Ruymgaart, A.; Hess, B. SHAKE parallelization. *The European Physical Journal Special Topics* **2011**, *200*, 211–223.

(16) Barth, E.; Kuczera, K.; Leimkuhler, B.; Skeel, R. D. Algorithms for constrained molecular dynamics. *Journal of Computational Chemistry* **1995**, *16*, 1192–1209.

(17) Gonnet, P.; Walther, J. H.; Koumoutsakos, P. Theta SHAKE: An extension to SHAKE for the explicit treatment of angular constraints. *Computer Physics Communications* **2009**, *180*, 360–364.

(18) Pechlaner, M.; Dorta, A. P.; Lin, Z.; Rusu, V. H.; van Gunsteren, W. F. A method to apply bond-angle constraints in molecular dynamics simulations. *Journal of Computational Chemistry* **2021**, *42*, 418–434.

(19) Pechlaner, M.; van Gunsteren, W. F. Algorithms to apply dihedral-angle constraints in molecular or stochastic dynamics simulations. *The Journal of Chemical Physics* **2020**, *152*.

(20) Dubbeldam, D.; Oxford, G. A.; Krishna, R.; Broadbelt, L. J.; Snurr, R. Q. Distance and angular holonomic constraints in molecular simulations. *The Journal of chemical physics* **2010**, *133*.

(21) Hayashi, Y.; Shiomi, J.; Morikawa, J.; Yoshida, R. RadonPy: automated physical property calculation using all-atom classical molecular dynamics simulations for polymer informatics. *npj Computational Materials* **2022**, *8*, 222.

(22) Huda, M. M.; Rai, N. Probing early-stage aggregation of low molecular weight gelator in an organic solvent. *The Journal of Physical Chemistry B* **2020**, *124*, 2277–2288.

(23) Ciulla, M. G.; Fontana, F.; Lorenzi, R.; Marchini, A.; Campone, L.; Sadeghi, E.; Paleari, A.; Sattin, S.; Gelain, F. Novel self-assembling cyclic peptides with reversible supramolecular nanostructures. *Materials Chemistry Frontiers* **2023**, *7*, 3680–3692.

(24) Wacha, A.; Varga, Z.; Beke-Somfai, T. Comparative Study of Molecular Mechanics Force Fields for $\beta$-Peptidic Foldamers: Folding and Self-Association. *Journal of Chemical Information and Modeling* **2023**, *63*, 3799–3813.

(25) Marrink, S. J.; Risselada, H. J.; Yefimov, S.; Tieleman, D. P.; De Vries, A. H. The MARTINI force field: coarse grained model for biomolecular simulations. *The Journal of Physical Chemistry B* **2007**, *111*, 7812–7824.

(26) Hammonds, K.; Heyes, D. Shadow Hamiltonian in classical NVE molecular dynamics simulations: A path to long time stability. *The Journal of Chemical Physics* **2020**, *152*.

(27) Ryckaert, J. P.; Ciccotti, G.; Berendsen, H. J. C. Numerical integration of the Cartesian equations of motion of a system with constraints: Molecular dynamics of n-alkanes. *Journal of Computational Physics* **1977**, *23*, 327–341.

(28) Hess, B.; Bekker, H.; Berendsen, H. J. C.; Fraaije, J. G. E. M. LINCS: A Linear

constraint solver for molecular simulations. *Journal of Computational Chemistry* **1997**, *18*, 1463–1472.

(29) Hess, B. P-LINCS: A parallel linear constraint solver for molecular simulation. *Journal of Chemical Theory and Computation* **2008**, *4*, 116–122.

(30) Verlet, L. Computer "experiments" on classical fluids. I. Thermodynamical properties of Lennard-Jones molecules. *Phys. Rev.* **1967**, *159*, 98–103.

(31) Hairer, E.; Wanner, G.; Lubich, C. *Geometric Numerical Integration*; Springer, 2006.

(32) Moré, J. J. Nonlinear Generalizations of Matrix Diagonal Dominance with Applications To Gauss-Seidel Iteration. *SIAM Journal on Numerical Analysis* **1972**, *9*, 357–378.

(33) Porshing, T. A. On the Rates of Convergence of Jacobi and Gauss-Seidel Methods for M-functions. *SIAM Journal on Numerical Analysis* **1971**, *8*, 575–582.

(34) López-Villellas, L.; Mikkelsen, C.; Galano, J.; Marco-Sola, S.; Alastruey, J.; Ibáñez Marín, P.; Moretó, M.; Sancho, J.; García-Risueño, P. Accurate and efficient constrained molecular dynamics of polymers using Newton's method and special purpose code. *Computer Physics Communications* **2023**, *288*, 108742.

(35) Phillips, J. C.; Braun, R.; Wang, W.; Gumbart, J.; Tajkhorshid, E.; Villa, E.; Chipot, C.; Skeel, R. D.; Kalé, L.; Schulten, K. Scalable Molecular Dynamics with NAMD. *Journal of Computational Chemistry* **2005**, *26*, 1781–1802.

(36) Phillips, J. C. et al. Scalable molecular dynamics on CPU and GPU architectures with NAMD. *The Journal of Chemical Physics* **2020**, *153*.

(37) Pearlman, D. A.; Case, D. A.; Caldwell, J. W.; Ross, W. R.; Cheatham III, T. E.; DeBolt, S.; Ferguson, D.; Seibel, G.; Kollman, P. AMBER, a computer program for applying molecular mechanics, normal mode analysis, Molecular Dynamics and free

energy calculations to elucidate the structures and energies of molecules. *Comp. Phys. Commun.* **1995**, *91*, 1–41.

(38) Brooks, B. R. et al. CHARMM: The biomolecular simulation program. *Journal of Computational Chemistry* **2009**, *30*, 1545–1615.

(39) Ortega, J. M.; Rheinboldt, W. C. *Iterative solution of nonlinear equations in several variables*; Computer science and applied mathematics; Academic Press: New York, 1970.

(40) Kelley, C. T. *Iterative methods for linear and nonlinear equations*; Frontiers in applied mathematics 16; Society for Industrial and Applied Mathematics: Philadelphia, 1995.

(41) Lou, G. Parallel methods for solving linear systems via overlapping decomposition. M.Sc. thesis, University of Illinois at Urbana-Champaign, 1989.

(42) Golub, G. H., Van Loan, C. F., Eds. *Matrix Computations*, 2nd ed.; The Johns Hopkins University Press: Baltimore and London, 1993.

(43) Karypis, G.; Kumar, V. Multilevelk-way partitioning scheme for irregular graphs. *Journal of Parallel and Distributed Computing* **1998**, *48*, 96–129.

(44) Amestoy, P. R.; Davis, T. A.; Duff, I. S. An approximate minimum degree ordering algorithm. *SIAM Journal on Matrix Analysis and Applications* **1996**, *17*, 886–905.

(45) Zlatev, Z.; Dimov, I.; Faragó, I.; Havasi, A. *Richardson Extrapolation: Practical Aspects and Applications*; De Gruyter, 2018.

(46) Kjelgaard Mikkelsen, C. C.; López-Villellas, L. The need for accuracy and smoothness in numerical simulations. Parallel Processing and Applied Mathematics, 15th International Conference PPAM 2024. 2025; pp 3–16.

(47) García-Risueño, P.; Ibáñez, P. E. A review of high performance computing foundations for scientists. *International Journal of Modern Physics C* **2012**, *23*, 1230001.

(48) Martin, C.; Richard, V.; Salem, M.; Hartley, R.; Mauguen, Y. Refinement and structural analysis of Barnase at 1.5 Å resolution. *Acta Crystallographica Section D: Biological Crystallography* **1999**, *55*, 386–398.

(49) Mackerell Jr., A. D.; Feig, M.; Brooks III, C. L. Extending the treatment of backbone energetics in protein force fields: Limitations of gas-phase quantum mechanics in reproducing protein conformational distributions in molecular dynamics simulations. *Journal of Computational Chemistry* **2004**, *25*, 1400–1415.

(50) Jorgensen, W. L.; Chandrasekhar, J.; Madura, J. D.; Impey, R. W.; Klein, M. L. Comparison of simple potential functions for simulating liquid water. *The Journal of Chemical Physics* **1983**, *79*, 926–935.

(51) Cotton, F. A.; Hazen, E. E.; Legg, M. J. Staphylococcal nuclease: Proposed mechanism of action based on structure of enzyme—thymidine 3', 5'-bisphosphate—calcium ion complex at 1.5-Å resolution. 1979; `http://dx.doi.org/10.1073/pnas.76.6.2551`.

(52) Douangamath, A.; Fearon, D.; Gehrtz, P.; Krojer, T.; Lukacik, P.; Owen, C. D.; Resnick, E.; Strain-Damerell, C.; Aimon, A., et al. Crystallographic and electrophilic fragment screening of the SARS-CoV-2 main protease. *Nature Communications* **2020**, *11*, 1–11.

(53) Núñez-Rojas, E.; Flores-Ruiz, H. M.; Alejandre, J. Molecular dynamics simulations to separate benzene from hydrocarbons using polar and ionic liquid solvents. *Journal of Molecular Liquids* **2018**, *249*, 591–599.

(54) Schmid, N.; Eichenberger, A. P.; Choutko, A.; Riniker, S.; Winger, M.; Mark, A. E.; van Gunsteren, W. F. Definition and testing of the GROMOS force-field versions 54A7 and 54B7. *European Biophysics Journal* **2011**, *40*, 843–856.

(55) Wu, B.; Mohideen, K.; Vasudevan, D.; Davey, C. A. Structural Insight into the Sequence Dependence of Nucleosome Positioning. *Structure* **2010**, *18*, 528–536.

(56) Tian, C.; Kasavajhala, K.; Belfon, K. A. A.; Raguette, L.; Huang, H.; Migues, A. N.; Bickel, J.; Wang, Y.; Pincay, J.; Wu, Q.; Simmerling, C. ff19SB: Amino-Acid-Specific Protein Backbone Parameters Trained against Quantum Mechanics Energy Surfaces in Solution. *Journal of Chemical Theory and Computation* **2020**, *16*, 528–552.

(57) Zgarbová, M.; Šponer, J.; Jurečka, P. Z-DNA as a Touchstone for Additive Empirical Force Fields and a Refinement of the Alpha/Gamma DNA Torsions for AMBER. *Journal of Chemical Theory and Computation* **2021**, *17*, 6292–6301.

(58) Izadi, S.; Anandakrishnan, R.; Onufriev, A. V. Building Water Models: A Different Approach. *The Journal of Physical Chemistry Letters* **2014**, *5*, 3863–3871.

(59) Li, P.; Song, L. F.; Merz, K. M. Systematic Parameterization of Monovalent Ions Employing the Nonbonded Model. *Journal of Chemical Theory and Computation* **2015**, *11*, 1645–1657.

(60) Sengupta, A.; Li, Z.; Song, L. F.; Li, P.; Merz, K. M. Parameterization of Monovalent Ions for the OPC3, OPC, TIP3P-FB, and TIP4P-FB Water Models. *Journal of Chemical Information and Modeling* **2021**, *61*, 869–880.

(61) Sui, H.; Han, B.-G.; Lee, J. K.; Walian, P.; Jap, B. K. Structural basis of water-specific transport through the AQP1 water channel. *Nature* **2001**, *414*, 872–878.

(62) Huang, J.; Rauscher, S.; Nawrocki, G.; Ran, T.; Feig, M.; de Groot, B. L.; Grubmüller, H.; MacKerell, A. D. J. CHARMM36m: an improved force field for folded and intrinsically disordered proteins. *Nature Methods* **2017**, *14*, 71–73.

(63) Huang, J.; MacKerell Jr, A. D. CHARMM36 all-atom additive protein force field: Validation based on comparison to NMR data. *Journal of Computational Chemistry* **2013**, *34*, 2135–2145.

(64) Kim, J.; Oh, J. H.; Kim, D. Recent advances in single-benzene-based fluorophores: Physicochemical properties and applications. *Organic & Biomolecular Chemistry* **2021**, *19*, 933–946.

(65) Levental, I.; Lyman, E. Regulation of membrane protein structure and function by their lipid nano-environment. *Nature Reviews Molecular Cell Biology* **2023**, *24*, 107–122.

(66) Jo, S.; Kim, T.; Iyer, V. G.; Im, W. CHARMM-GUI: A web-based graphical user interface for CHARMM. *Journal of Computational Chemistry* **2008**, *29*, 1859–1865.

(67) Lee, J. et al. CHARMM-GUI Input Generator for NAMD, GROMACS, AMBER, OpenMM, and CHARMM/OpenMM Simulations Using the CHARMM36 Additive Force Field. *Biophysical Journal* **2015**, *12*, 405–413.

(68) Wu, E. L.; Cheng, X.; Jo, S.; Rui, H.; Song, K. C.; Dávila-Contreras, E. M.; Qi, Y.; Lee, J.; Monje-Galvan, V.; Venable, R. M.; Klauda, J. B.; Im, W. CHARMM-GUI *Membrane Builder* toward realistic biological membrane simulations. *Journal of Computational Chemistry* **2014**, *35*, 1997–2004.

(69) Jo, S.; Kim, T.; Im, W. Automated builder and database of protein/membrane complexes for molecular dynamics simulations. *PLoS ONE* **2007**, *2*, e880.

(70) Jorgensen, W. L.; Chandrasekhar, J.; Madura, J. D.; Impey, R. W.; Klein, M. L. Comparison of simple potential functions for simulating liquid water. *The Journal of Chemical Physics* **1983**, *79*, 926–935.

(71) Izadi, S.; Anandakrishnan, R.; Onufriev, A. V. Building water models: a different approach. *The Journal of Physical Chemistry Letters* **2014**, *5*, 3863–3871.

(72) Páll, S.; Hess, B. A flexible algorithm for calculating pair interactions on SIMD architectures. *Computer Physics Communications* **2013**, *184*, 2641–2650.

(73) Essmann, U.; Perera, L.; Berkowitz, M. L.; Darden, T.; Lee, H.; Pedersen, L. G. A smooth particle mesh Ewald method. *The Journal of Chemical Physics* **1995**, *103*, 8577–8593.

(74) Haug, E. J.; Arora, J. S.; Matsui, K. A steepest-descent method for optimization of mechanical systems. *Journal of Optimization Theory and Applications* **1976**, *19*, 401–424.

(75) Bussi, G.; Zykova-Timan, T.; Parrinello, M. Isothermal-isobaric Molecular Dynamics using stochastic velocity rescaling. *The Journal of Chemical Physics* **2009**, *130*, 074101.

(76) Berendsen, H. J. C.; Postma, J. P. M.; van Gunsteren, W. F.; DiNola, A.; Haak, J. R. Molecular dynamics with coupling to an external bath. *The Journal of Chemical Physics* **1984**, *81*, 3684–3690.

(77) Parrinello, M.; Rahman, A. Polymorphic transitions in single crystals: A new molecular dynamics method. *Journal of Applied Physics.* **1981**, *52*, 7182–7190.

(78) Galano-Frutos, J. J.; Sancho, J. Accurate Calculation of Barnase and SNase Folding Energetics Using Short Molecular Dynamics Simulations and an Atomistic Model of the Unfolded Ensemble: Evaluation of Force Fields and Water Models. *Journal of Chemical Information and Modeling* **2019**, *59*, 4350–4360.

(79) Galano-Frutos, J. J.; Nerín-Fonz, F.; Sancho, J. Calculation of Protein Folding Thermodynamics Using Molecular Dynamics Simulations. *Journal of Chemical Information and Modeling* **2023**, *63*, 7791–7806.

(80) Estrada, J.; Bernadó, P.; Blackledge, M.; Sancho, J. ProtSA: a web application for calculating sequence specific protein solvent accessibilities in the unfolded ensemble. *BMC Bioinformatics* **2009**, *10*, 104.

(81) Becktel, W. J.; Schellman, J. A. Protein stability curves. *Biopolymers* **1987**, *26*, 1859–1877.
